# Recent advances in activated carbon magnetic composites (ACMCs): synthesis, characterization, performance as efficient adsorbents and other applications

**DOI:** 10.3389/fchem.2026.1931555

**Published:** 2026-09-28

**Authors:** Zakaria Al-Qodah, Asma Eskhan, Ibrahim Suleiman, Zaid A. Al-Anber, Ahmad T. Shawaqfeh

**Affiliations:** Department of Chemical Engineering, Faculty of Engineering Technology, Al-Balqa Applied University, Amman, Jordan

**Keywords:** activated carbon, magnetic activated carbon, magnetic composites, modeling and optimization of ACMCs processes, preparation and characterization of ACMCs, catalysis of ACMCs

## Abstract

Activated carbon (AC) is widely recognized material for its high porosity, expansive specific surface area, and an exceptional adsorption capacity. To explore these traits, the impregnation of magnetic particles into AC matrix has emerged as a key innovative strategy to produce composites with enhanced performance as adsorbents or catalysts. The literature survey indicates a surge in publications post-2010, to produce adsorbents of high capacity and selectivity as dominant subject areas. The resulting AC magnetic composites (ACMCs) simplifies the recovery via an external magnetic field and extend ACMCs to other application beyond traditional water treatment. The applications of ACMCs have primarily centered on adsorption, to remove both organic and inorganic pollutants, these composites have shown remarkable performance as catalysts, biocatalyst support, and magnetic solid-phase extraction. In most applications, iron and iron oxides have served as the main magnetic materials. This review provides a comprehensive analysis of the diverse synthesis pathways used to fabricate ACMCs, alongside the essential characterization techniques required to evaluate their structural and chemical properties. Furthermore, it summarizes the kinetic and isotherm models applied to ACMCs systems, while exploring how statistical tools and machine learning methods are being deployed to optimize operational parameters. This unpreceded integration of structural, kinetic, and optimization offers a definitive roadmap of ACMCs literature since the year 2000, serving as a vital cornerstone for future research.

## Graphical Abstract

**Figure:**
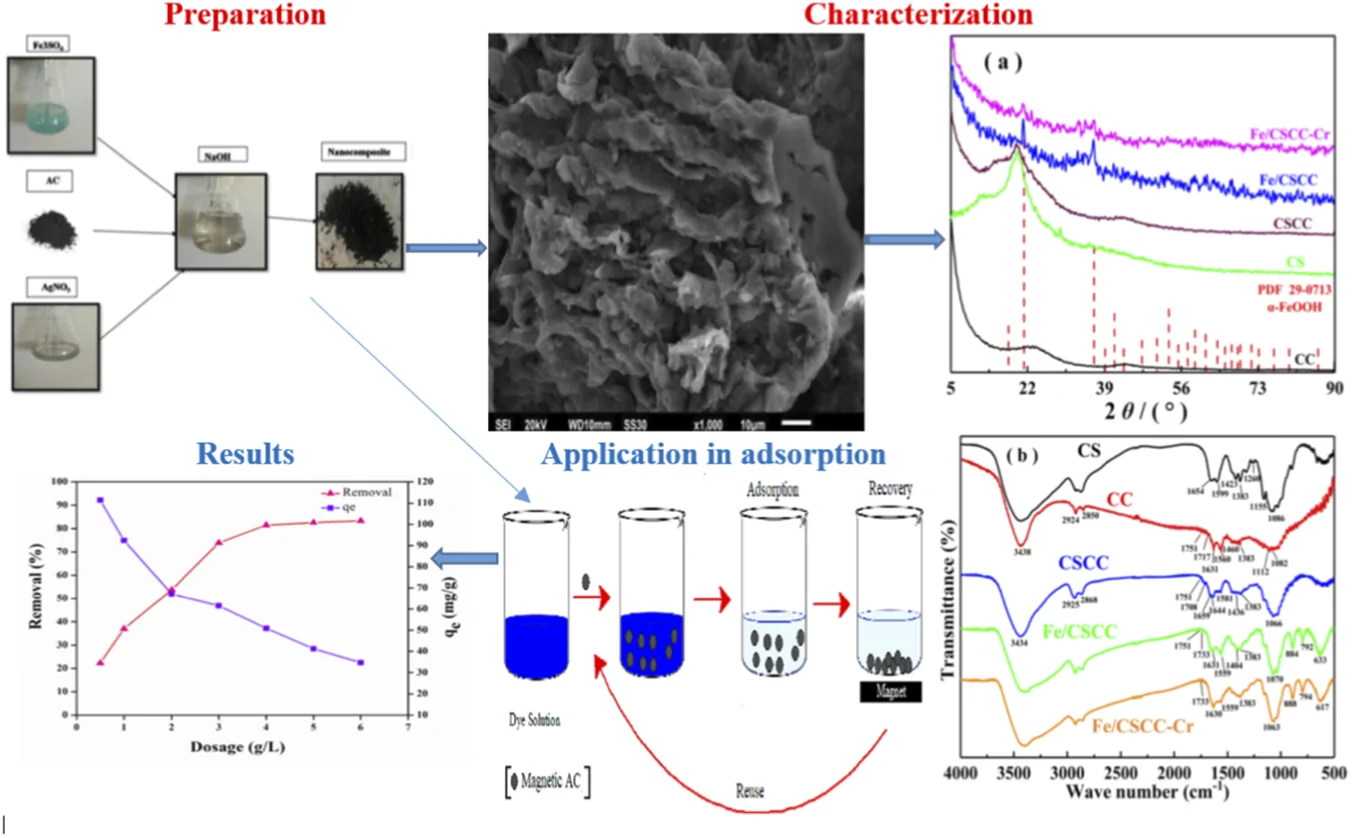

## 1 Introduction

Ativated carbon (AC) is a very important chemical of unique structure, of a relatively high surface area and porosity (Safarik et al., 2012). In addition, its abundance, low cost, and ease of large-scale production gives it distinguished characteristics as an adsorbent, and as a catalyst support in many chemical, industrial and environmental applications (Al-Qodah and Lafi, 2001; Song et al., 2023; Sun et al., 2018). However, the issue of AC separation from their liquid media for possible regeneration or management as a waste remains a series technical challenge, especially if the AC size is in the Nano, micro or fine levels (Lee and Ahmad Zaini, 2022; Moussa et al., 2021; Oh et al., 2015; Zadeh and Jafari, 2024).

Accordingly, AC should be modified using suitable approaches to enhance its separation performance to overtake the previous challenges (Furlan et al., 2017; Kakavandi et al., 2015; Moussa et al., 2021; Niculescu et al., 2024; Thue et al., 2020; Zarei and Sinapour, 2015). One of these approaches brings light to adding magnetic properties to AC by the formation of a suitable AC composite (Al-Qodah and Al-Shannag, 2006; Sharma et al., 2022). The ability to separate and recover spent magnetic materials after use is an important characteristic of such AC composites. This separation can be performed by applying a suitable magnetic field (Azam et al., 2022; Safarik et al., 2012). Activated carbon magnetic composites (ACMCs) are being prepared in many procedures that incorporates a suitable magnetic material of Nano or micro size in the AC structure (Song et al., 2023; Al-Qodah et al., 2015; Krishna et al., 2017; Al-Qodah et al., 2023). In addition, ACMCs have been utilized in many applications including adsorption of organic and inorganic pollutants, catalyst and biocatalyst supports and in other limited applications (Sharma et al., 2022). In all of these applications, the results have confirmed that these composites have high potentials as efficient separable and generable adsorbents, catalysts and catalysts support (Al-Qodah and Al-Hassan, 2000; Al-Qodah and Lafi, 2001; Humadi and Mohammed, 2026; Iles et al., 2025).

The literature survey concerning the application of ACMCs indicated that its application is relatively recent. It showed that most of research papers on ACMCs appeared since the year 2000. Table 1 shows the field of ACMCs applications and the number of researches in each applications.

**TABLE 1:** Number and field of application of researches on ACMCs since the year 2000.

Years	Adsorbent for inorganic pollutants		Adsorbent of organic pollutants		Support for biocatalysts		Catalyst		Others	
	Magnetic material	Ref.	Magnetic material	Ref.	Magnetic material	Ref.	Magnetic material	Ref.	Magnetic material	Ref.
2000–2004	Fe_3_O_4_	Phanapavudhikul et al. (2003)	γ-Fe_3_O_4_	Al-Qodah (2000b), Al-Qodah et al. (2001)	Iron	Al-Qodah (2000a), Al-Qodah and Al-Hassan (2000)	​	(Al-Qodah and Lafi, 2001)	Fe_3_O_4_	Al-Qodah (2000b), Al-Qodah et al. (2001)
2005–2009	Fe_3_O_4_	Vaughan and Reed (2005)	Iron oxide, MnFe_2_O_4_, FeCl_3_·FeCl_2_·Fe_3_O_4_	Al-Busoul et al. (2008), Al-Qodah and Al-Busoul (2000), Al-Qodah et al. (2001), Al‐Qodah and Al‐Shannag (2007), Al‐Qodah and Al‐Shannag (2007), Oliveira et al. (2002), Phanapavudhikul et al. (2003), Yang et al. (2008)	Iron	​	​	​	Iron	Al-Qodah and Al-Shannag (2006)
2010–2014	Iron oxide, Fe_3_O_4_	Yao et al. (2014), Zhang et al. (2018)	CuFe_2_O_4_, Fe_3_O_4_	Ngarmkam et al. (2011), Singh et al. (2010)	Iron, Fe_3_O_4_	Zheng et al. (2012)	CuFe_2_O_4_, Ferrites, Fe_3_O_4_	Kakavandi et al. (2015), Oh et al. (2015), Reza et al. (2015), Zarei and Sinapour (2015)	Iron	​
2015–2019	CoFe_2_O_4_, Fe, NiCl_2_, Fe_3_O_4_	Allwar et al. (2020), Chen et al. (2018), Foroutan et al. (2018), Jain et al. (2018), Juang et al. (2018), Le et al. (2019), Liu et al. (2015), Saleh et al. (2017), Seeharaj et al. (2019), Thue et al. (2020)	Fe_3_O_4_/Fe^0^, FeSO_4_, Fe, MgFe_2_O_4_, Iron oxide, NiCl_2_, Fe_3_O_4_	Altıntıg et al. (2017), Fröhlich et al. (2019), Kakavandi et al. (2015), Niculescu et al. (2024), Oh et al. (2015), Strachowski et al. (2017), Subha et al. (2018), Zarei and Sinapour (2015)	Fe_3_O_4_	Al-Qodah et al. (2015)	Fe_3_O_4_	Gholinejad et al. (2016)	Iron oxide, Fe_3_O_4_	Abellán-Martín et al. (2023), Al-Busoul et al. (2008), Anjana and Chandra (2023), Furlan et al. (2017), Mohd Jamaludin et al. (2023), Niculescu et al. (2024), Rodrigues et al. (2020), Rodríguez et al. (2015), Sabaa et al. (2022), Seeharaj et al. (2019), Zhao et al. (2019)
2020–2024	Iron, NiFe_2_O_4_, γ-Fe_3_O_4,_ Iron oxide, Fe_3_O_4_ ZnCl_2_-FeCl_3_	Ahmad et al. (2021), Al-Qodah et al. (2023), Altamer et al. (2024), Arul et al. (2023), Farhana et al. (2022), Gheitasi et al. (2022), Hashemi and Soleimani (2025), Khalil et al. (2022), Liu et al. (2022), Liu et al. (2023), Mohamed et al. (2024), Moussa et al. (2021), Ngan et al. (2024), Ramya et al. (2022), Scalera et al. (2025), Takmil et al. (2020), Thue et al. (2020), Zhang et al. (2022)	Fe(NO_3_)_3_· PbFe_2_O_4_, FeSO_4_, Iron, Fe0–Cu Lead ferrite, Fe_3_O_4_ NiFe_2_O_4_, FeCl_3_, iron scrap, Ni_0.4_Fe_2_.6O_4_	Lee and Ahmad Zaini (2022), Zadeh and Jafari (2024), Wan et al. (2020), Le et al. (2019), Wang et al. (2021), Louis et al. (2023), Manousi et al. (2021), Abdollahzadeh et al. (2022), Foroutan et al. (2022), Yu et al. (2022), Barzegarzadeh and Amini-Fazl (2025b), Mirzaee (2022), Khaleghi et al. (2022), Spessato et al. (2020), Mirzaee and Sartaj (2022), Rashtbari et al. (2022), Abdel-Aziz et al. (2023), Kartika Santosa and Rizkiana (2023), Allahkarami et al. (2023), Bouider and Rida (2024), Kord Mostafapour et al. (2024), Deivayanai et al. (2024), Lv et al. (2024), Alam et al. (2024), Thue et al. (2024), Khalil et al. (2024), Molina-Jordá (2022), Zeng et al. (2022), Moosavi et al. (2020), Yanti et al. (2023), Saadi et al. (2024), Meng et al. (2025), Mohammadi et al. (2020), Takmil et al. (2020), Shin et al. (2021), Zazouli et al. (2021), Tsoutsa et al. (2023), Romero et al. (2026), Kodandoor et al. (2026), Yang et al. (2023)	​	Al-Qodah et al. (2017a), Matveeva and Bronstein (2021), Saire-Saire et al. (2020), Santos et al. (2024), Torres et al. (2018), Wang et al. (2024)	MFe_2_O_4_, CoFe_2_O_4_ MgFe_2_O_4_ FeCl_3_, Fe_3_O_4_	Faheem et al. (2024), Hemalatha et al. (2024), Lataf et al. (2024), Schneider et al. (2020), Song et al. (2023), Zazouli et al. (2021)	​	​
2025–2026	Iron, NiFe_2_O_4_	[51=54, Dar and Kurella (2025), Goren (2026), Sharififard (2026), Shewatatek et al. (2025), Sun et al. (2025)]	FeSO_4_, Iron, Fe0–Cu Lead ferrite	Ahmed et al. (2026), Asadzadeh et al. (2025), Iles et al. (2025), Jiang et al. (2022), Kodandoor et al. (2026), Li et al. (2026), Lima et al. (2025), Phanapavudhikul et al. (2003), Rinovian et al. (2026), Romero et al. (2026), Tran et al. (2021)	​	​	​	​	​	​
Total	​	40	​	68	​	10	​	10	​	14

According to the information shown in Table 1, the literature concerning magnetic ACMCs contains more than 141 papers published since the year 2000. These researches confirmed that magnetic composites are superior to non-magnetic composites in many properties especially in their ease of separation. The distribution of the published papers in this field on the application and years of publication is shown in Figure 1.

**FIGURE 1:**
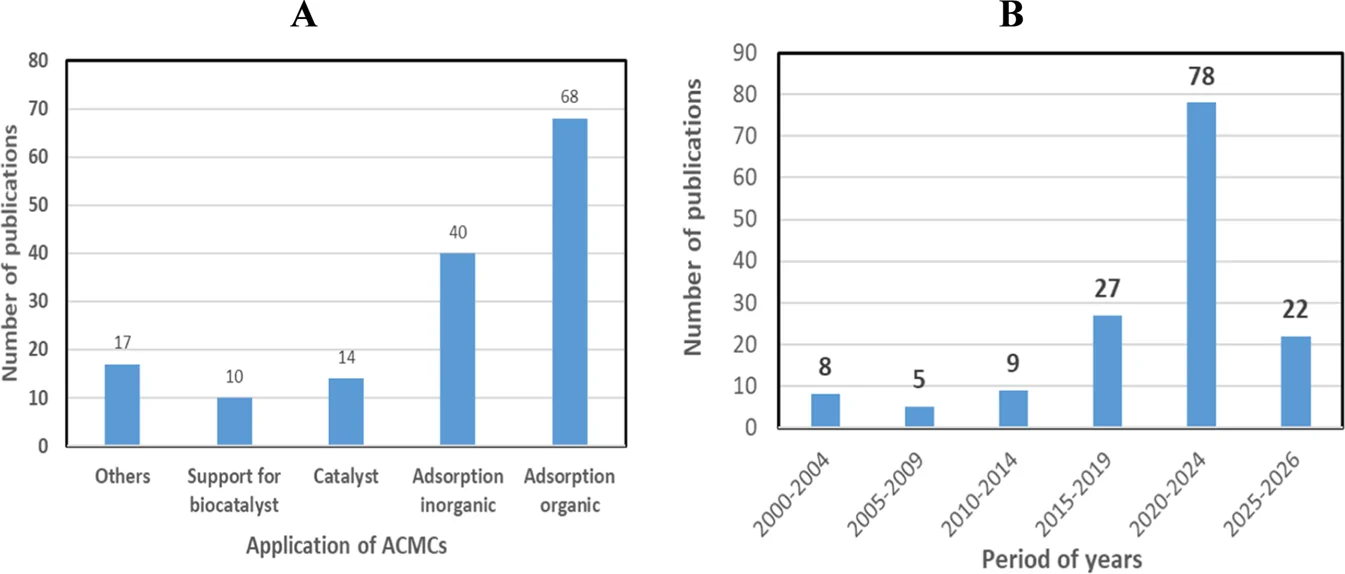
**(A)** The distribution of the published papers on ACMCs since the year 2000, **(B)** Different applications.

It is evident from Figure 1A that is about 68, 40, 17, 14 and 17 published papers, considered organic materials adsorption, inorganic materials adsorption, catalysts, support for biocatalysts and other applications, respectively. Moreover, Figure 1B indicates that the number of publications per 5 years increases significantly in the period 2020 to 2024, where 52% of the publications have been cited. For this reason, reviewing and analyzing theses researches and highlighting the important characteristics, advancements and challenge of ACMCs becomes a priority. In addition, suitable recommendations for future works are needed in these reviews to give researchers important insights about the present status and the possible future developments and innovations (Al-Qodah et al., 2017a; Safarik et al., 2012; Shewatatek et al., 2025; Sivashankar et al., 2014; Song et al., 2023).

Several reviews concerning the performance of magnetic composites and their different applications have been published in the last 2 decades. Table 2 summarizes the main features and conclusions of these reviews for general magnetic composites and ACMCS.

**TABLE 2:** The information about main reviews concerning magnetic composites and applications since the year 2000.

A- General magnetic composites				
Ref.	Title	Intention	Discussed parameters	Main conclusions
Niculescu et al. (2024)	An Updated Overview of Magnetic Composites for Water Decontamination	Adsorption of pollutants	Presents the latest advancements of magnetic composites	High adsorption capacity for industrial wastewater pollutants
Sharma et al. (2022)	Recent advances in magnetic composites as adsorbents for wastewater remediation	Adsorption of metal ions	Composites and nanocomposites magnetic adsorbents for pollutants	Composite materials have high adsorption capacity
Sivashankar et al. (2014)	Magnetic composite an environmental super adsorbent for dye sequestration	Adsorption of dyes	Adsorption performance, preparation, characteristics	MC have high adsorption efficiency, ease of regeneration
Al-Qodah et al. (2017a)	Immobilized enzymes bioreactors utilizing a magnetic field: A review	Enzymes support	Adsorption mechanism, preparation, characteristics	MC have high adsorption efficiency, ease of regeneration
Al-Qodah et al. (2017b)	On the performance of immobilized cell bioreactors utilizing a magnetic field	Cells support	Kinetic and mass transfer models of immobilized cells in MSFBs	Improve the stability of biocatalysts. No downstream recovery steps
Wu et al. (2020)	Multifunctional magnetic soft composites: a review	Robotics/biomedical	Performance of multifunctional response of soft MC	The development of magnetic soft composites faces challenges
Mourdikoudis et al. (2021)	Magnetic Nanoparticle Composites: Synergistic Effects and Applications	Nanoparticle Composites	Nanocomposites magnetics, support separable catalysts	MC and NC has pioneering roles as catalysis, and biomedicine
Zeng et al. (2022)	Adsorption of heavy metal ions in water by surface functionalized magnetic composites: a review	Adsorption of metal ions	The performance of MC composite as adsorbents for heavy metal ions	MA have ease of recovery, high adsorptive capacity, high selectivity
Deivasigamani et al. (2025)	Magnetic Nanoparticles: Synthesis, characterization and application based on environmental Perspective	Adsorption of toxic pollutants	Synthesizing, applications and understanding of carbon-based magnetic nanoparticles (MNP)	effective and economical Nano magnetic adsorbents for toxic pollutants in aqueous systems
B- Activated carbon magnetic composites (ACMCs)				
Safarik et al. (2012)	Magnetically Responsive Activated Carbons for Bio - and Environmental Applications	Adsorption, immobilization	Preparation and applications AM derivatives in chemistry, biotechnology, and environment	Efficient adsorbents suitable for immobilization of bioactive enzymes or cells
Song et al. (2023)	Recent advances in persulfate activation by magnetic ferrite-carbon composites for organic contaminants degradation: Role of carbon materials and environmental application	Persulfate (PS) to degrade organics	The mechanism for PS activation by MFC composite materials and the degradation of organic pollutants in MFC/PS systems	Couples spinel ferrite with AC improves the efficiency of PS activation and solves the separation problem
Azam et al. (2022)	A review on activated modifications for the treatment of wastewater containing anionic dyes	Adsorption of anionic dyes	Analyzing the ACs functional groups, properties	Modified-AC’s has high adsorption capacity, low cost and tunable oxygen functionality
Rafli et al. (2021)	Magnetite-Activated Carbon Composite to Reduce Pollutant: Review	Adsorption of metals ions	Properties of ACMCS along with magnetite benefits and applications	AC synthesis from PE plastic waste as efficient adsorbent for air heavy metals
Sudarsan et al. (2026)	Recent Advances in MnFe_2_O_4_/Activated Carbon Composites for Environmental and Energy Applications: A Comprehensive Review on Synthesis, Characterization, and Performance	Adsorption and photocatalytic activities	The magnetic and catalytic activity Structural characterizations adsorption and photocatalysis Scalability, regeneration, and real wastewater applications	High adsorption and photocatalytic performances Synergistic mechanisms enhance pollutant removal Crystalline MnFe_2_O_4_ uniformly dispersed within the AC matrices
Amili et al. (2026)	Sustainable adsorption technologies for textile dye removal: Advances in biomass-derived and magnetically modified activated carbons	Dye adsorption	Dye-polluted wastewater treatment Particular focus on adsorption Factors affecting adsorption an regeneration	Adsorption is one of the most effective and versatile methods for removing textile dyes Adsorbents magnetic properties enhance separation and recovery

Table 2 indicates that most of the published reviews considered general magnetic composites and not those of AC, such as the reviews of the numbers: (Al-Qodah et al., 2017a; Niculescu et al., 2024; Sharma et al., 2022; Sivashankar et al., 2014), and (Al-Qodah et al., 2017b; Deivasigamani et al., 2025; Mourdikoudis et al., 2021; Wang et al., 2022; Wu et al., 2020; Zeng et al., 2022). On the other hand, only few reviews concerning AC or its allotropes such magnetic composites have been published in the same period (Azam et al., 2022; El Meziani et al., 2025; Rafli et al., 2021; Safarik et al., 2012; Song et al., 2023). As can be seen in Table 2 part a, most of these reviews have not considered ACMCs and they are limited to general magnetic composites or to non-magnetic AC composites. In the other hand, Table 2 part b summarizes five short reviews as the following: Safarik et al. (2012), summarized in their short review the procedures used to convert activated carbons into to some magnetic derivatives. In addition, it summarized ACMCs applications in biotechnology, medicine, analytical chemistry and environment. Song et al. (2023), reviewed the recent advances in persulfate activation by magnetic ferrite-carbon (MFC) composite materials for organic pollutants degradation in water. Azam et al. (2022), critically analyzed AC modification researches conducted to enhance their capacity for effective removal of anionic dyes from wastewater. In addition, the discussed the corresponding AC modification techniques, and properties. Rafli et al. (2021), reviewed researches dealing with AC preparation from plastic-magnetite nanoparticles to be used as an adsorbent of heavy metals ions found in air. Finally, Agasti et al. (El Meziani et al., 2025), presented in their reviewed the main results and prospects in carbon nanotube based magnetic composites used for contaminants removal. Based on the available published reviews so far, it is clear that there are no published comprehensive reviews concerning ACMCs researches. For this reason, a comprehensive review for researches dealing with ACMCS will be of vital importance to such researchers due to their exponential increase in the numbers and applications. Accordingly, this review systematically summarizes the latest research progress and advancement in ACMCs since the year 2000. In addition, a comprehensive focus on their types, characteristics, methods of preparation, and applications represent parts of this review. Other composites of carbon allotropes such as biochar, graphene and carbon nanotubes will not be part of this review. Moreover, this review will give insights on the present challenges, and future prospects in the use and application of ACMCs as an efficient adsorbent for organic and inorganic pollutants from wastewater.

## 2 Main preparation processes for ACMCs composites

The preparation of ACMCs has been performed using various processes; each of them has some advantages and drawbacks. The used processes are classified into eight categories: (1) precipitation, (2) co-precipitation, (3) thermal conversion, (4) hydrothermal (5) polymer- and structure-directed synthesis, (6) bimetallic and *in-situ* reduction, (7) advanced hybrid and variant demotion methods.

These applied processes for ACMCs preparations have different chemical principles, thermal treatments, and structural design in adding the magnetic materials in the carbon supports. The following sections discuss the detail procedures of each preparation process; summarize the main results in the relative studies, and a comparative discussion for their performance.

### 2.1 Precipitation method

The precipitation method can be used to synthesize modified ACMCs nanocomposites. This procedure consists of two main steps: (i) The preparation of the ACMCs support and (ii) an optional surface activation or functionalization. Acid oxidation pretreatment is usually applied to the AC, commonly to introduce oxygen-containing functional groups to enhance hydrophilic its properties. Ferric salts such as ${\text{FeCl}}_{3}$ , then dissolved in the aqueous solution to combine with the AC in the suspension. After that, the addition of a base, such as NaOH or ${\text{NH}}_{4}\text{OH}$ , leads to the formation of iron hydroxides precipitates, which transform upon aging and controlled heating, into iron oxides nanoparticles, such as ${\text{Fe}}_{3}O_{4}$ or ${\gamma -\text{Fe}}_{2}O_{3}$ . These nanoparticles firmly deposited on the AC matrix. After separation of the formed ACMCs solid, it is washed several times to eliminate residual ions. It is then dried to produce a stable ACMCs. Some studies included additional steps, such as calcination, the addition of secondary agents such as urea and ethanol. Other studies perform surface modifications, to enhance the functional and structural characteristics of the prepared ACMCs composite.

This preparation method was performed by several studies. Zarei and Sinapour (Strachowski et al., 2017) used ${\text{FeCl}}_{3}$ precipitation process to prepare ACMCs to remove azide ions. The process demonstrated an effective adsorption of ${\text{FeCl}}_{3}$ followed by subsequent precipitation at 70 °C. Finally, the precipitate was then washed and dried. Altintig et al. (Subha et al., 2018) applied a similar process for the adsorption of methylene blue (MB), using ferric salts as magnetic precursors. Furthermore, Oliveira et al. (Jain et al., 2018) used ${\text{FeCl}}_{3}$ -derived precipitation for the production of AC-iron oxide composites to remove some pollutant. In addition, Allwar et al. (Manousi et al., 2021) enhanced this process by combining waste biomass palm shell-derived AC with ${\text{FeCl}}_{3}$ precipitation. Ethanol/water functional agent were incorporated to enhance the surface properties of the prepared ACMCs. Louis et al. (Luu et al., 2022) incorporated urea as a functional additive, and then applied calcination to the composite at 350 °C to promote crystallinity and stability. This process resulted in Fe-AC magnetic composite with tailored properties. Finally, the Ahmed et al., 2025 and Zhang et al., 2022 demonstrated the effectiveness of the ACMCs prepared by this process to remove heavy metals ions, such as Pb (II) and Cd (II) as model pollutants.

It is evident from the above discussion that the precipitation method demonstrate a balance between synthesis simplicity and functional efficacy. Although these materials may not reach the phase uniformity or magnetic strength of co-precipitation-derived ${\text{Fe}}_{3}O_{4}$ composites, the method offers a rapid, adaptable, and scalable approach to ACMCs, allowing for further optimization through additives, hydrothermal treatments, or calcination steps.

### 2.2 Co-precipitation method

One of the common process applied for preparing ACMCs is co-precipitation, which is characterized by its operational simplicity, reproducibility. In addition, this process can produce well-dispersed magnetic nanoparticles such as ${\text{Fe}}_{3}O_{4}$ or ${\gamma -\text{Fe}}_{2}O_{3}$ in the carbon support. Co-precipitation differs from conventional precipitation method, which uses a single ferric salt precursor (e.g., ${\text{FeCl}}_{3}$ ). In co-precipitation technique, both ${\text{Fe}}^{2+}$ and ${\text{Fe}}^{3+}$ salts are simultaneously dissolved in an aqueous solution at a specified molar ratio such as ${\text{Fe}}^{2+}:{\text{Fe}}^{3+}=1:2$ . Precipitation is induced by the gradual addition of a base, such as NaOH or ${\text{NH}}_{3}$ , under vigorous stirring, typically within an inert atmosphere, such as $N_{2}$ or Ar, and at moderate heating conditions of 60 − 90 °C. In this step, Fe^+2^ and Fe^+3^ ions undergo hydrolysis and co-precipitation, resulting in the formation ${\text{Fe}\left(\text{OH}\right)}_{2}$ and ${\text{Fe}\left(\text{OH}\right)}_{3}$ mixture, which then converts into magnetite nanoparticles.

A suspension of AC in water is introduced to the reaction mixture prior to or during the precipitation step. This facilitates the *in-situ* deposition of magnetic nanoparticles onto its surface. The black suspension is generally aged for several hours to improve particle crystallization and adhesion, subsequently undergoing filtration, repeated washing with distilled water and ethanol to eliminate unreacted ions, and drying at moderate temperatures (80 − 105 °C). Post-treatments, including calcination and surface modification, are sometimes implemented to refine the structural and magnetic properties. This process produces composites featuring uniformly distributed magnetite nanoparticles anchored to the carbon matrix, which exhibit high adsorption capacity and strong magnetic recoverability.

Multiple studies have applied this method with various AC precursors. (Kakavandi et al., 2015), (Furlan et al., 2017), (Sivaprakash et al., 2017), (Krishna et al., 2017), (Oliveira et al., 2002), (Al-Qodah and Al-Busoul, 2000), (Yang et al., 2008), (Luo and Zhang, 2009), (Juang et al., 2018), (Manousi et al., 2021), (Hammouda et al., 2022), (Faheem et al., 2024), (Altamer et al., 2024), (Ngan et al., 2024), (Zeng et al., 2022), (Hashemi and Soleimani, 2025) and (Wang et al., 2022) used powdered or granular AC in conjunction with ${\text{FeCl}}_{2}$ and ${\text{FeCl}}_{3}$ as precursors. Sun et al. (2018) conducted the deposition of ${\text{Fe}}_{3}O_{4}$ nanoparticles onto AC fibers under comparable conditions. Activated carbon derived from biomass has been utilized in various studies. Ngarmkam et al. (Zhu et al., 2011) employed palm shell, Yu et al. (Spessato et al., 2020) and Ben Hammouda (Bouider and Rida, 2024) used sludge-derived activated carbon, Jamaludin et al. (2023) utilized coconut shell, and Khaleghi et al. (Kord Mostafapour et al., 2024) prepared AC from date seeds. In addition to pristine magnetite composites, dopants have been utilized to modify the properties of magnetic anisotropic composites, including spinel ferrites and oxide hybrids. Subsequently, Foroutan et al. (Louis et al., 2023) synthesized ${\text{CoFe}}_{2}O_{4}$ /AC for the removal of Cr(VI), whereas Allahkarami et al. (Liu et al., 2022) prepared ${\text{PbFe}}_{2}O_{4}$ /AC composites. Additionally, Foroutan et al. (Allahkarami et al., 2022) reported on ZnO/ ${\text{Fe}}_{3}O_{4}$ /AC hybrids for the adsorption of methyl violet 2B dye, while Mirshafiee et al. synthesized nanoparticles of ${\text{Cu}}_{2}O$ / ${\text{Fe}}_{3}O_{4}$ supported on coconut shells activated carbon.

The co-precipitation method can be tailored through post-treatment functionalization to enhance the effectiveness of ACMCs. Gheitasi et al. (Kartika Santosa and Rizkiana, 2023) modified the resulting ${\text{Fe}}_{3}O_{4}$ /AC by introducing thiol groups, thereby creating a functionalized sorbent that exhibits high selectivity for Hg(II) ions.

### 2.3 Thermal conversion methods

Thermal conversion methods, including pyrolysis, calcination, carbothermic reduction, and microwave heating, constitute a thorough set of techniques for the production of ACMCs. In contrast to precipitation-based methods that depend on aqueous chemistry, thermal approaches utilize controlled high-temperature processing to carbonize the feedstock while concurrently incorporating magnetic phases, including magnetite ( ${\text{Fe}}_{3}O_{4}$ ), maghemite ( ${\gamma -\text{Fe}}_{2}O_{3}$ ), or spinel ferrites ( ${\text{MFe}}_{2}O_{4}$ , where M=Ni,Co,Mg,Pb ). These methods exhibit significant versatility, demonstrating compatibility with both commercial AC and a diverse range of biomass precursors. The resulting composites exhibit high structural stability, tunable porosity, and a strong magnetic response. Thermal processing generally entails higher energy consumption compared to precipitation or co-precipitation. It necessitates careful control of parameters, including temperature, heating rate, and furnace atmosphere, to optimize pore development alongside effective magnetic loading.

The precursor material, either AC or biomass feedstock, is initially impregnated with an iron salt solution, typically FeCl_3_, Fe(NO_3_)_3_ or mixed metal nitrates, to achieve uniform dispersion of metal species. Following the drying process, the impregnated solid undergoes thermal treatment under controlled conditions, generally within the temperature range of 500 − 900 °C, contingent upon the targeted carbonization and magnetic phase. The procedure can be conducted in a muffle furnace using air or inert gases ( $N_{2}$ , Ar) for calcination or pyrolysis, respectively, or by utilizing microwave heating for efficient and rapid conversion. Heating results in the carbonization of the organic matrix, while the metal precursors decompose and reduce, producing iron oxide or ferrite nanoparticles within the carbon framework. Subsequent to thermal treatment, the product undergoes cooling, washing to eliminate unreacted salts and loosely bound residues, and drying to yield a stable ACMCs composite. Some studies incorporate additional steps, such as activation with ${\text{ZnCl}}_{2}$ or KOH, or post-calcination modifications, to enhance surface area and functionality.

Several sub-routes were reported within Pyrolysis and Calcination method. The one-step activation-magnetization process, carried out by Lee and Zaini (2022), integrates chemical activation agents like $\text{Zn}{\text{Cl}}_{2}$ with iron precursors ( $\text{Fe}{\text{Cl}}_{3}$ or mixed Fe salts) and employs pyrolysis, facilitating the simultaneous occurrence of carbonization, activation, and magnetization. This route minimizes processing steps and produces highly porous ACMCs; however, it is essential to carefully remove residual Zn species. Biomass pyrolysis-derived ACMCs constitute a significant route, utilizing feedstock’s including tucumã fruit shells (Thue et al., 2020), waste tires (Mirzaee, 2022), brachychiton populneus fruit crust (Molina-Jordá, 2022), corn stover (Al-Qodah et al., 2017b), pomegranate peel (Mourdikoudis et al., 2021), and cork (Deivasigamani et al., 2025). The precursors are impregnated with Fe salts before pyrolysis, leading to the creation of magnetically responsive carbons and the utilization of many waste materials. Their attractiveness is rooted in scalability and sustainability; however, variability in feedstock composition may hinder reproducibility.

Specialized carbothermic and composite methods, (Baker et al., 2007), (Al-Busoul et al., 2008), (Le et al., 2019), (Wang et al., 2021), (Mohamed et al., 2024), and (Arul et al., 2023) utilize high-temperature reduction to integrate iron oxides or create mesoporosity. Combining AC with clay or conducting carbothermic reduction under controlled conditions can produce mesoporous ACMCs with improved textural properties; however, these methods typically require increased energy input and more strict control over reaction atmospheres. Calcination-based ferrite composites, reported by Hemalatha et al. (Salama et al., 2024) and Thue et al. (Wu et al., 2020), concentrate on the synthesis of spinel structures, including $\text{Ni}{\text{Fe}}_{2}O_{4}$ , and ${\text{Fe}}_{3}O_{4}$ , embedded within a carbon matrix. The precursor, such as microcrystalline cellulose impregnated with ${\text{FeCl}}_{3}$ , is subjected to pyrolysis or calcination at temperatures ranging from 600 − 700 °C to induce crystallization of the ferrite phase. This process results in materials exhibiting enhanced structural stability and magnetic responsiveness, though it reduces surface area. Microwave-assisted pyrolysis, carried out by Lataf et al. (Furlan et al., 2017), is a contemporary method that employs microwave irradiation to efficiently carbonize hardwood precursors treated with Fe salts. This method markedly decreases reaction times and energy consumption relative to traditional furnaces; however, scalability continues to pose a challenge.

Thermal and calcination-based methods are notable for their versatility in feedstock selection, the ability to integrate multiple synthesis steps, and the potential to produce robust and magnetically strong composites. Thermal methods require greater energy input than aqueous precipitation or co-precipitation; however, they typically offer improved structural integrity, greater compatibility with biomass precursors, and increased durability of magnetic phases in challenging operating conditions.

### 2.4 Hydrothermal and solvothermal method

Hydrothermal and solvothermal synthesis methods offer a unique approach for the production of magnetic activated carbon, characterized by their capacity to function under sealed, high-pressure conditions that facilitate the uniform crystallization of magnetic nanoparticles. In contrast to precipitation methods that depend on ambient aqueous reactions or pyrolysis techniques necessitating high-temperature furnaces, hydrothermal synthesis entails the suspension of AC (or biomass-derived precursors) with iron salts in an aqueous solution, subsequently treated in a Teflon-lined autoclave at elevated temperatures (generally 150 − 250 °C). The regulated temperature and pressure conditions promote the uniform distribution of ${\text{Fe}}_{3}O_{4}$ or spinel ferrite nanoparticles within the carbon matrix, enhancing their adhesion and long-term stability relative to traditional wet impregnation methods. Solvothermal variants enhance this method by replacing water with organic solvents (e.g., ethanol, ethylene glycol), allowing for improved control over particle size, morphology, and surface functionalization.

Initially, AC or a biomass precursor is dispersed in an aqueous or organic solution that contains iron salts, including ${\text{FeCl}}_{3}.6H_{2}O$ , ${\text{Fe}\left({\text{NO}}_{3}\right)}_{3}$ , or mixed metal nitrates. The suspension undergoes ultrasonication or stirring to ensure uniform dispersion prior to being placed in a sealed autoclave reactor. The system undergoes heating to 150 − 250 °C for a duration of 6–24 h, facilitating the hydrolysis and crystallization of iron precursors, resulting in the formation of well-dispersed iron oxide or ferrite nanoparticles anchored to the carbon matrix. Upon reaching room temperature, the solid product is obtained through filtration or centrifugation, washed carefully with distilled water and ethanol to eliminate residual ions and solvents, and subsequently dried at 80 − 105 °C. A subsequent calcination step (300 − 500 °C) may be employed to enhance crystallinity or modify magnetic properties.

This category is exemplified by several representative studies. Sivaprakash et al. (Hardian et al., 2021) utilized a hydrothermal post-treatment following initial precipitation to enhance the stabilization of ${\text{Fe}}_{3}O_{4}$ on biomass-AC, resulting in improved crystallinity and magnetic stability. Wan et al. (Le et al., 2019) modified this method by applying hydrothermal treatment to sludge-derived activated carbon, followed by calcination, leading to integrating waste valorization with the addition of a durable magnetic phase. Chandra (Ramya et al., 2022) employed a solvothermal-like method to incorporate $\text{Co}{\text{Fe}}_{2}O_{4}$ nanoparticles into activated carbon, resulting in a composite that exhibits improved spinel ferrite characteristics for magnetic recovery and catalytic performance.

In comparison to precipitation or co-precipitation methods, hydrothermal and solvothermal approaches provide notable benefits in the synthesis of well-dispersed, strongly bonded, and crystallographically uniform magnetic phases. Nonetheless, these methods necessitate extended reaction times, pressurized reactors, and increased energy input, rendering them more complex than ambient precipitation techniques. Their primary strength is the production of composites that exhibit enhanced durability, phase purity, and functional performance, especially in contexts demanding robust cycling stability or advanced catalytic applications.

### 2.5 Polymer- and structure-directed synthesis methods

Polymer- and structure-directed synthesis methods offer an alternative approach for the production of ACMCS, highlighting the importance of morphological control and functional versatility compared to traditional precipitation or pyrolysis techniques. This method involves the integration of AC into polymeric or structured matrices, including cellulose, polyHIPEs, or hydrogels, which serve as scaffolds for the consistent deposition of magnetic nanoparticles. The geometry and properties of the polymer support, such as beads, foams, and gels, determine the final architecture of the composite, resulting in materials that exhibit improved mechanical integrity, enhanced processability, and customized sorption performance. In contrast to hydrothermal or thermal methods that primarily emphasize crystallinity and phase control of ${\text{Fe}}_{3}O_{4}$ , polymer-directed techniques concentrate on the engineered design of the sorbent’s overall structure to meet application-specific requirements.

Activated carbon, either in powdered or granulated form, is incorporated into a polymer precursor solution or emulsion, such as cellulose acetate, polyacrylamide, or polyHIPE. Iron salts, including ${\text{FeCl}}_{3}.6H_{2}O$ and ${\text{FeSO}}_{4}.7H_{2}O$ , are subsequently incorporated into the matrix, where they diffuse and adhere to the polymeric framework. The precipitation of iron oxides is generally initiated by the addition of a base (such as NaOH or ${\text{NH}}_{4}\text{OH}$ ) or through *in situ* reduction, resulting in the formation of ${\text{Fe}}_{3}O_{4}$ nanoparticles that are either entrapped or anchored within the polymer-carbon structure. The composite is subsequently stabilized through crosslinking, gelation, or curing, contingent upon the polymer employed. The product is subjected to washing and drying to eliminate unreacted species. In certain instances, calcination or solvent exchange may be employed to enhance structural integrity or adjust porosity. The resulting ACMCs composites integrate the adsorptive capacity of AC with the formability and stability of polymer structures, rendering them appropriate for dye removal, wastewater treatment, and reusable adsorbents.

Luo and Zhang (Ngarmkam et al., 2011) detailed the synthesis of magnetic cellulose beads incorporating activated carbon, wherein ${\text{Fe}}_{3}O_{4}$ nanoparticles were integrated into a cellulose-AC matrix to create spherical, reusable adsorbent beads with improved handling characteristics. Seeharaj et al. (2019) utilized a polyHIPE template to incorporate AC and iron oxides into a porous polymer-derived structure. This approach produced lightweight, mechanically strong composites featuring high internal surface area and adjustable pore structures, thereby enhancing the applicability of ACMCs in areas where structural integrity and reusability are as critical as adsorption efficiency.

The primary benefit of polymer/structured composites is their customized morphology, straightforward separation, and suitability for specific applications, including column packing, catalysis, and water treatment in dynamic flow environments. Nonetheless, they may exhibit a reduced overall carbon surface area in comparison to pure AC-based composites, and the presence of polymer residues could influence thermal or chemical stability. These systems illustrate the potential of integrating polymer chemistry with magnetic carbons to develop advanced functional composites that surpass the capabilities of traditional powdery ACMCs materials.

### 2.6 Bimetallic and *in-situ* reduction methods

Bimetallic and *in-situ* reduction methods serve as a targeted strategy for the synthesis of ACMCs, aimed at integrating magnetic properties with redox activity. In contrast to traditional precipitation or co-precipitation methods that predominantly produce ${\text{Fe}}_{3}O_{4}$ or ${\gamma -\text{Fe}}_{2}O_{3}$ phases, these approaches integrate zero-valent metals (such as ${\text{Fe}}^{0}$ , ${\text{Cu}}^{0}$ , ${\text{Ag}}^{0}$ ) into the carbon matrix via reductive treatments, typically employing ${\text{NaBH}}_{4}$ or environmentally friendly reductants. The resulting composites integrate the strong magnetic separability of Fe oxides with the catalytic or antimicrobial properties of the second metal, rendering them highly suitable for advanced water treatment, pollutant degradation, and antimicrobial applications.

Generally, activated carbon is initially impregnated or suspended in an aqueous solution containing iron precursors ( ${\text{Fe}}^{2+}$ / ${\text{Fe}}^{3+}$ salts), which are subsequently precipitated to form ${\text{Fe}}_{3}O_{4}$ nanoparticles on the carbon matrix. A solution of a second metal salt (e.g., ${\text{Cu}}^{2+}$ , ${\text{Ag}}^{+}$ ) is subsequently introduced, followed by a controlled addition of ${\text{NaBH}}_{4}$ , as a reducing agent, under vigorous stirring. The reduction step converts dissolved ions into zero-valent nanoparticles ( ${\text{Fe}}^{0}$ , ${\text{Cu}}^{0}$ , ${\text{Ag}}^{0}$ ), which are deposited *in situ* with the iron oxides. Following reduction, the composite is generally separated, subjected to repeated washing with distilled H_2_O and C_2_H_5_OH, and subsequently dried at moderate temperatures (80 − 105 °C). In certain instances, the procedure may incorporate an extra stabilization step, such as freeze-drying or calcination, to improve particle stability and mitigate oxidation. The final product integrates magnetic functionality with improved catalytic or antimicrobial performance, facilitating its application in various reactive environmental contexts.

A range of studies has been conducted, highlighting the diversity present in bimetallic and *in-situ* reduction approaches. Mirshafiee et al. (Khaleghi et al., 2022) describes the development of AC/ ${\text{Fe}}_{3}O_{4}+{\text{Cu}}_{2}O$ composites through the integration of copper oxides into a magnetized carbon framework, resulting in a bifunctional sorbent that exhibits improved adsorption and catalytic performance. Liu et al. (Allahkarami et al., 2023) utilized a sequential approach in which ${\text{Fe}}^{2+}/{\text{Fe}}^{3+}$ salts were initially co-precipitated onto activated carbon, followed by reduction with ${\text{NaBH}}_{4}$ to produce nanoscale zero-valent iron (nZVI), and subsequently modified with silver nanoparticles. This resulted in a bimetallic AC/nZVI-Ag composite exhibiting significant reactivity towards contaminants and facilitating easy magnetic recovery. Rashtbari et al. (Ngan et al., 2024) employed a green synthesis method, integrating ${\text{Fe}}^{0}$ and Cu nanoparticles with AC without the use of harsh chemical reductants, thereby presenting an environmentally sustainable alternative. Abdel-Aziz (Scalera et al., 2025) reported the incorporation of ${\text{Fe}}^{0}-\text{Cu}$ onto fava bean-derived activated carbon, demonstrating the versatility of biomass precursors in supporting bimetallic systems.

The efficacy of *in-situ* reduction methods is attributed to their capacity to synergistically integrate adsorption with catalytic degradation or antimicrobial activity, surpassing the passive removal mechanisms characteristic of traditional ACMCs. Implementation can be complex, necessitating high control of reduction steps to prevent particle aggregation, and may encounter stability issues due to the propensity of ${\text{Fe}}^{0}$ or ${\text{Cu}}^{0}$ to oxidize over time. Notwithstanding these limitations, these composites constitute a significant category of multifunctional ACMCs with potential applications in next-generation environmental remediation technologies.

### 2.7 Advanced hybrid methods

Metal–organic frameworks MOF, graphene, and encapsulated-metal hybrid approaches constitute an advanced category of routes for the preparation of ACMCs, highlighting nanoscale engineering and multifunctionality. In contrast to precipitation, pyrolysis, or hydrothermal methods that primarily emphasize the direct deposition of iron oxides on activated carbon, these approaches utilize high-surface-area supports, including graphene oxide (GO), metal–organic frameworks (MOFs), or encapsulation strategies to reach a precise control over particle morphology, distribution, and interfacial chemistry. The hybrids produced typically integrate the adsorption capacity of carbon with the catalytic, electronic, or structural advantages of advanced nanomaterials, resulting in composites that extend beyond conventional pollutant sorption to include catalytic and electronic applications.

In general, ACundergoes functionalization or is combined with advanced support materials, such as graphene oxide sheets, pre-synthesized MOFs, or polymer/oxide encapsulation matrices. Iron precursors, specifically ${\text{Fe}}^{3+}$ or mixed ${\text{Fe}}^{2+}/{\text{Fe}}^{3+}$ ^+^ salts, are subsequently added to the hybrid suspension. This is followed by precipitation or solvothermal treatment to facilitate the anchoring of ${\text{Fe}}_{3}O_{4}$ or spinel ferrite nanoparticles onto the carbon-support interface. In MOF-based systems, iron ions can be incorporated directly during the assembly of the MOF and later carbonized at high temperatures (500 − 900 °C) to produce a porous, conductive Fe-C framework. In methods utilizing graphene or encapsulation, ${\text{Fe}}_{3}O_{4}$ nanoparticles are generally produced through *in-situ* reduction or co-precipitation, which facilitates their immobilization within the layered or encapsulated framework. Following synthesis, the composites undergo washing, drying, and may be subjected to calcination or annealing to enhance crystallinity and stability. These procedures produce multifunctional composites that integrate magnetic separability with customized catalytic, electronic, or adsorption properties, distinguishing them from traditional ACMCs materials.

Shi and Ye (Barzegarzadeh and Amini-Fazl, 2025a) detailed the development of magnetite-graphene oxide composites, in which ${\text{Fe}}_{3}O_{4}$ nanoparticles were affixed to GO sheets, resulting in a high surface area, magnetically separable adsorbent characterized by improved dispersibility. Strachowski et al. (Amili et al., 2026) utilized waste polyethylene terephthalate (PET) as a carbon precursor, resulting in the production of Fe@C nanostructures encapsulated in carbon, thus integrating waste valorization with advanced Nano structuring techniques. Ma et al. (Mirzaee and Sartaj, 2022) illustrated the conversion of MOFs combined with wood scaffolds into magnetic hydrophilic composites, employing a distinctive “MOF-to-wood” approach that utilized the hierarchical porosity of wood alongside the customized chemistry of MOFs. Wei et al. (Anjana and Chandra, 2023) introduced graphene-encapsulated ferrites, where ferrite nanoparticles are enveloped in thin graphene shells. This configuration stabilizes the nanoparticles against leaching and improves their electron transport properties, which is particularly advantageous for catalytic and energy-related applications.

The primary benefit of these hybrid strategies is their capacity to integrate various functional components adsorption, magnetism, catalysis, and structural control into a single material. Nevertheless, their synthesis is typically more intricate, frequently necessitating multi-step processing, specialized precursors, or controlled environments. Although this approach incurs higher costs and limits scalability relative to simpler methods like precipitation or pyrolysis, these hybrid systems illustrate the potential of emerging materials, such as graphene and MOFs, to advance the design of multifunctional and high-performance materials for ACMCs applications.

### 2.8 Variant deposition

Variant deposition encompasses specialized techniques used in composite material preparation. It involves either intentionally depositing distinct matrix and reinforcement materials in varying gradients or addressing the natural compositional variations that occur when co-depositing multi-phase materials in manufacturing.

### 2.9 Comparison between ACMCs preparation methods

Various synthesis strategies have been utilized for the preparation of magnetic ACMCs, each with specific advantages, limitations, and applications. Table 3 presents a comprehensive summary of the primary preparation methods for comparison purposes. The table presents the essential features, common applications, and the comparative advantages and disadvantages of each approach. This summary highlights the variety of methods available and aids in determining the most appropriate technique for a specific application context. In addition, preparing ACMCs involves balancing functional porosity with sufficient magnetic response. Below is a quantitative comparison of the different synthesis methods across the specific surface area (SSA) and saturation magnetization (SM).

**TABLE 3:** Comparative summary of preparation methods for magnetic activated carbon.

Preparation method	Main characteristics				Advantages	Disadvantages	Main references
	General	SSA m^2^/g	SM emu/g	Temp. ^o^C			​
Co-precipitation	Dissolves ${\text{Fe}}^{2+}$ / ${\text{Fe}}^{3+}$ salts; precipitates ${\text{Fe}}_{3}O_{4}$ / ${\gamma -\text{Fe}}_{2}O_{3}$ directly on AC in a basic medium	400–1100	20–45	60–80	High magnetization, controlled stoichiometry	Requires inert atmosphere, sensitivity to ${\text{Fe}}^{2+}$ / ${\text{Fe}}^{3+}$ ratio	Kakavandi et al. (2015), Zarei and Sinapour (2015), Furlan et al. (2017), Krishna et al. (2017), Al-Qodah and Al-Busoul (2000), Luo and Zhang (2009), Zhu et al. (2011), Reza et al. (2015), Juang et al. (2018), Khalil et al. (2024)
Thermal conversion	Biomass or AC precursors impregnated with Fe salts; high temperature treatment (conventional/microwave)	600–1350	25–60	400–900	Strong magnetic phases, stable composites, compatible with diverse feed stocks	Energy intensive, may reduce surface area, requires temperature control	Al-Busoul et al. (2008), Al-Qodah et al. (2017b), Arul et al. (2023), Deivasigamani et al. (2025), Lataf et al. (2024), Lee and Ahmad Zaini (2022), Mirzaee (2022), Molina-Jordá (2022), Mourdikoudis et al. (2021), Salama et al. (2024), Thue et al. (2020), Wang et al. (2021), Wu et al. (2020)
Hydrothermal/Solvothermal	Fe salts + AC treated in sealed autoclave at 150 − 250 °C; promotes a uniform ${\text{Fe}}_{3}O_{4}$ / ${\text{Fe}}_{2}O_{3}$ crystallization	250–850	30–70	15–260	Good dispersion of nanoparticles, strong adhesion, crystalline phases	Long reaction times, costly equipment, higher energy demand	Hardian et al. (2021), Le et al. (2019), Ramya et al. (2022)
Polymer/Structure-Directed	Incorporates AC into beads, gels, foams (e.g., cellulose, HIPE); Fe oxides dispersed within polymer matrix	500–1400	15–40	60–80 aging 500–800 pyrolysis	Good handling, reusability, tailored morphology	Lower surface area, complex synthesis, polymer residues	Ngarmkam et al. (2011), Seeharaj et al. (2019)
Bimetallic/*In-situ* Reduction	Co-precipitated ${\text{Fe}}_{3}O_{4}$ further reduced ( $\text{NaB}H_{4}$ , green reductants) with secondary metals (Cu, Ag, nZVI)	400–1000	40–90	300–800	Synergistic adsorption + catalytic degradation, high reactivity	More complex synthesis, particle aggregation, ${\text{Fe}}^{0}$ / ${\text{Cu}}^{0}$ oxidation risk	Allahkarami et al. (2023), Khaleghi et al. (2022), Ngan et al. (2024), Scalera et al. (2025)
Advanced Hybrids	Uses GO, MOFs, or encapsulation to support Fe oxides/ferrites; nanoscale engineered composites	350–1200	2050	200–1000 Rapid heating via microwaves	Multifunctionality (adsorption, catalysis, conductivity), tunable	Multi-step, costly precursors, low scalability	Amili et al. (2026), Anjana and Chandra (2023), Barzegarzadeh and Amini-Fazl (2025a), Mirzaee and Sartaj (2022)

Table 3 clearly indicates that precipitation, co-precipitation and thermal conversion processes are the predominant techniques used for the preparation of ACMCs. This predominance can be attributed to their simplicity, low cost, scalability, and the capacity to produce stable magnetite or maghemite nanoparticles with satisfactory magnetic properties. While precipitation is simple, low-cost, scalable, and adaptable to AC, co-precipitation exhibit high magnetization, and controlled stoichiometry. In addition, thermal conversion processes produce strong magnetic phases, stable composites, and compatible with diverse feedstock. On the other hand, advanced preparation methods for ACMCs like hydrothermal, Solvothermal, polymer/structure-Directed, bimetallic/*in-situ* reduction or hybrid strategies offer improved functionality and customized structural characteristics. However, they typically entail greater energy demands, extended processing durations, or more intricate synthesis procedures. Accordingly, the selection of the preparation method is significantly determined by the intended application, weighing economic feasibility and process simplicity against the requirements for advanced performance or multifunctionality.

## 3 Characterization of ACMCs

Activated carbon magnetic composites (ACMCs) characterization is an important step after their preparation. The main objectives of this step are focused on understanding and evaluating the properties and behavior of the prepared materials to ensure that the procedure is suitable to give the required composites needed for specific performance, design, and certain application requirements. This will help in understanding and optimizing the suitable conditions for the preparation of the material’s magnetic, structural, and physical properties to ensure it performs effectively in its intended application as shown in Figures 2, 3.

**FIGURE 2:**
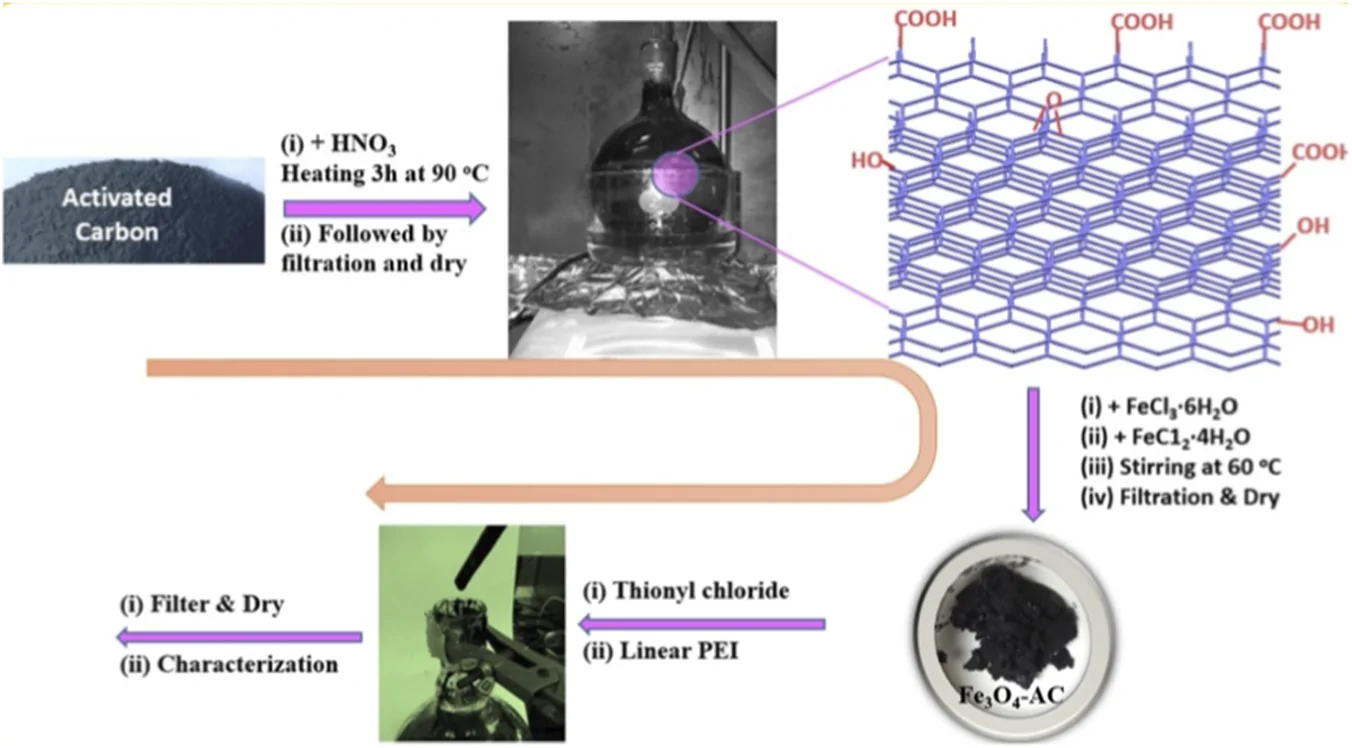
Illustration for the preparation of PAF magnetic sorbent (Saleh et al., 2017).

**FIGURE 3:**
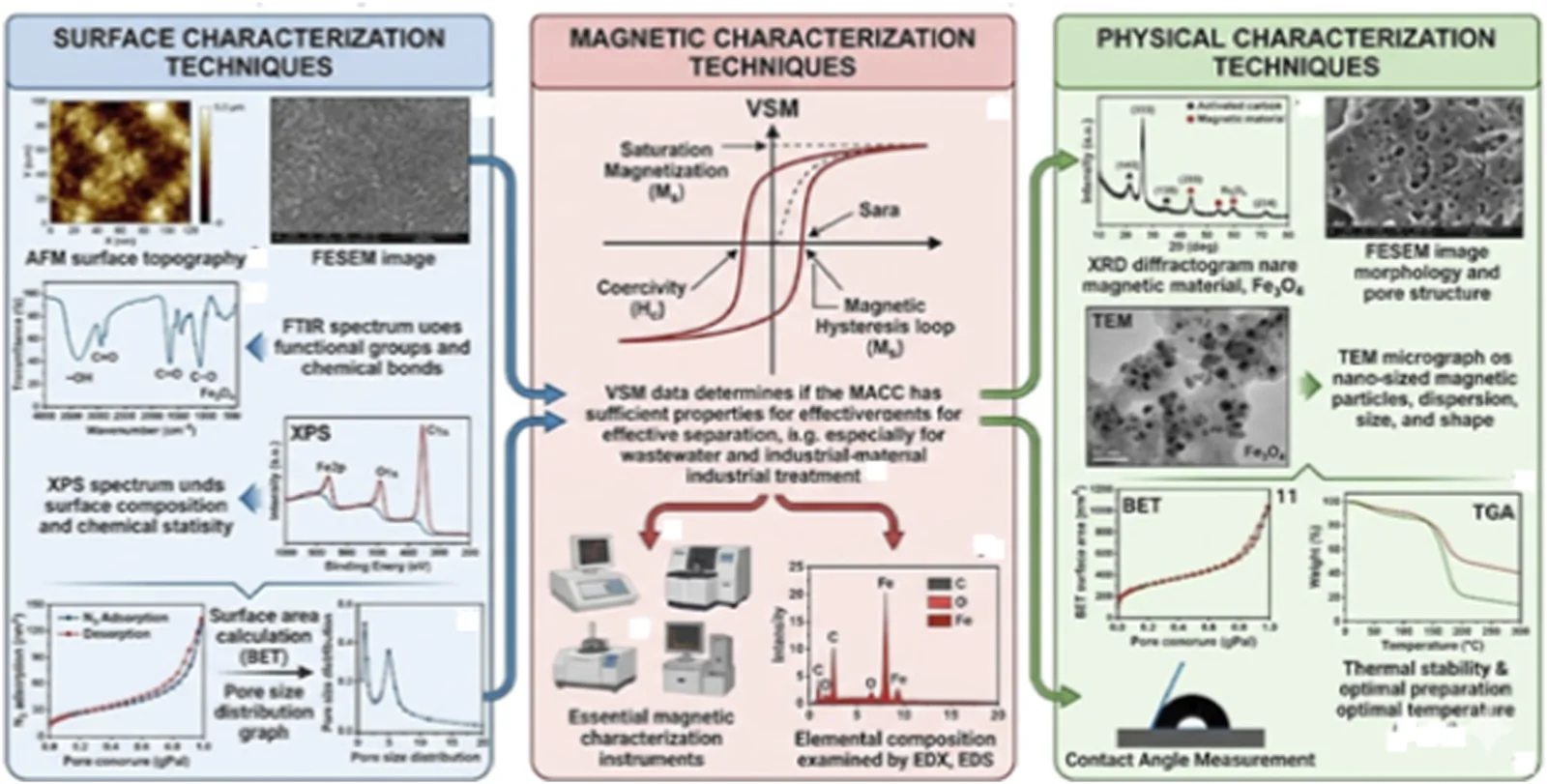
The primary classification areas of characterization techniques of ACMCs (Source: Gemini).

The characterization techniques can be classified into three main categories as the following:

1. Surface Characterization Techniques, which include the following methods: Fourier Transform Infrared Spectroscopy (FTIR), Scanning Electron Microscopy (SEM), Field Emission Scanning Electron Microscopy (FESEM), Energy Dispersive X-ray spectroscopy (EDX), Energy Dispersive Spectroscopy (EDS), Brunauer-Emmett-Teller (BET), Ultraviolet–visible spectroscopy (UV-VIS), X-ray Photoelectron Spectroscopy {XPS), N_2_ adsorption-desorption isotherms, Raman spectroscopy, Boehm Titration and the Point of Zero Charge (pHpzc).
2. Magnetic Characterization Techniques, which include the following methods: Vibrating Sample Magnetometry (VSM), Atomic Absorption Spectroscopy (AAS),
3. Physical Characterization Techniques, which include the following methods: X-ray Diffraction (XRD), Transmission Electron Microscopy (TEM), Thermogravimetric Analysis (TGA), Contact Angle Measurement and Atomic Force Microscopy (AFM).

The intention of this review is to highlights the main advantages of these methods rather than describing their operation. More information can be found in references that are more specialized. Table 4 presents research articles of the most widely used characterization techniques and their specific findings in the context of ACMCs.

**TABLE 4:** Some of the most applied characterization processes for Magnetic ACMCs.

Technique	Method	Main characteristics and findings	References
Surface	FTIR	• Detect functional groups and magnetic nanoparticles. Clarify structure and vibrational bands. Reveal O_2_ groups • (-OH, -COOH, C=O) and Fe_3_O_4_ incorporation	Abdel-Aziz et al. (2023), Alam et al. (2024), Alameri et al. (2024), Allahkarami et al. (2022), Allwar et al. (2020), Arul et al. (2023), Barzegarzadeh and Amini-Fazl (2025b), Chen et al. (2018), Farhana et al. (2022), Fitri et al. (2024), Foroutan et al. (2018), Gheitasi et al. (2022), Hammouda et al. (2022), Jain et al. (2018), Juang et al. (2018), Kartika Santosa and Rizkiana (2023), Khaleghi et al. (2022), Kord Mostafapour et al. (2024), Le et al. (2019), Lee and Ahmad Zaini (2022), Liu et al. (2022), Louis et al. (2023), Luu et al. (2022), Lv et al. (2024), Manousi et al. (2021), Mirzaee (2022), Moussa et al. (2021), Ngan et al. (2024), Oh et al. (2015), Ramya et al. (2022), Rashtbari et al. (2022), Rinovian et al. (2026), Saleh et al. (2017), Saning et al. (2024), Scalera et al. (2025), Spessato et al. (2020), Subha et al. (2018), Sudarsan et al. (2026), Thue et al. (2024), Wan et al. (2020), Wang et al. (2021), Wen et al. (2022), Zadeh and Jafari (2024), Zhang et al. (2022)
	SEM	• Intricate images of surface structure pore characteristics, and dispersion of magnetic particles within the AC matrix	Abdel-Aziz et al. (2023), Abdollahzadeh et al. (2022), Ahmad et al. (2021), Alameri et al. (2024), Allahkarami et al. (2022), Allahkarami et al. (2023), Allwar et al. (2020), Amili et al. (2026), Barzegarzadeh and Amini-Fazl (2025b), Bouider and Rida (2024), Chen et al. (2018), Fitri et al. (2024), Foroutan et al. (2018), Foroutan et al. (2022), Gheitasi et al. (2022), Hammouda et al. (2022), Hemalatha et al. (2024), Jain et al. (2018), Juang et al. (2018), Kakavandi et al. (2015), Khalil et al. (2024), Kord Mostafapour et al. (2024), Le et al. (2019), Lee and Ahmad Zaini (2022), Liu et al. (2022), Louis et al. (2023), Lv et al. (2024), Manousi et al. (2021), Mirzaee and Sartaj (2022), Moussa et al. (2021), Oh et al. (2015), Rinovian et al. (2026), Saleh et al. (2017), Saning et al. (2024), Scalera et al. (2025), Seeharaj et al. (2019), Spessato et al. (2020), Subha et al. (2018), Sudarsan et al. (2026), Wan et al. (2020), Wang et al. (2021), Yu et al. (2022), Zadeh and Jafari (2024), Zhang et al. (2022)
​	FESEM	• High-resolution characterization of surface topography, pore structure, and insertion/dispersion of magnetic nanoparticles	Alam et al. (2024), Farhana et al. (2022), Hemalatha et al. (2024), Kartika Santosa and Rizkiana (2023), Khaleghi et al. (2022), Luu et al. (2022), Mirzaee (2022), Ngan et al. (2024), Ramya et al. (2022), Saning et al. (2024), Schneider et al. (2020), Thue et al. (2024), Wen et al. (2022)
	EDX	• Qualitative and quantitative elemental composition (C, O, Fe, secondary dopants) before/after adsorption	Ahmad et al. (2021), Alameri et al. (2024), Allahkarami et al. (2022), Allwar et al. (2020), Arul et al. (2023), Farhana et al. (2022), Fitri et al. (2024), Foroutan et al. (2018), Foroutan et al. (2022), Hammouda et al. (2022), Kakavandi et al. (2015), Khaleghi et al. (2022), Kord Mostafapour et al. (2024), Liu et al. (2022), Louis et al. (2023), Luu et al. (2022), Manousi et al. (2021), Mirzaee (2022), Oh et al. (2015), Ramya et al. (2022), Rashtbari et al. (2022), Wang et al. (2021), Wen et al. (2022), Zadeh and Jafari (2024)
	EDS	• Confirm presence and uniform spatial distribution of magnetic elements within the AC matrix	Abdel-Aziz et al. (2023), Allahkarami et al. (2023), Bouider and Rida (2024), Chen et al. (2018), Gheitasi et al. (2022), Saning et al. (2024), Subha et al. (2018), Sudarsan et al. (2026), Thue et al. (2024), Zhang et al. (2022)
	BET	• Determine specific surface area, pore volume, and pore size distribution (micropores, mesopores)	Alam et al. (2024), Allahkarami et al. (2022), Allwar et al. (2020), Arul et al. (2023), Bouider and Rida (2024), Chen et al. (2018), Deivayanai et al. (2024), Foroutan et al. (2018), Gheitasi et al. (2022), Hammouda et al. (2022), Hemalatha et al. (2024), Jain et al. (2018), Juang et al. (2018), Kakavandi et al. (2015), Khaleghi et al. (2022), Khalil et al. (2024), Kord Mostafapour et al. (2024), Le et al. (2019), Lee and Ahmad Zaini (2022), Liu et al. (2022), Luu et al. (2022), Mirzaee and Sartaj (2022), Moussa et al. (2021), Ngan et al. (2024), Oh et al. (2015), Rinovian et al. (2026), Saning et al. (2024), Spessato et al. (2020), Subha et al. (2018), Thue et al. (2024), Wang et al. (2021), Wen et al. (2022), Yu et al. (2022), Zadeh and Jafari (2024), Zhang et al. (2022)
	UV-VIS	• Measure light absorbance before and after interaction to evaluate equilibrium concentration and isotherm models	Alameri et al. (2024), Fitri et al. (2024), Foroutan et al. (2022), Rashtbari et al. (2022), Saleh et al. (2017), Subha et al. (2018), Sudarsan et al. (2026), Wan et al. (2020), Yu et al. (2022)
	XPS	• Near-surface elemental composition, chemical/oxidation states, and functional group identification	Allahkarami et al. (2023), Gheitasi et al. (2022), Le et al. (2019), Mirzaee and Sartaj (2022), Schneider et al. (2020), Spessato et al. (2020), Sudarsan et al. (2026)
​	N_2_ adsorption desorption	• Measures surface area and pore size distribution to assess adsorption capacity	Abdel-Aziz et al. (2023), Allahkarami et al. (2022), Allahkarami et al. (2023), Amili et al. (2026), Kartika Santosa and Rizkiana (2023), Rashtbari et al. (2022)
	Raman spectroscopy	• Evaluates molecular vibrational modes, graphitic structural order, and lattice defects (ID/IG ratio)	Allahkarami et al. (2022), Allahkarami et al. (2023), Allwar et al. (2020), Amili et al. (2026), Lv et al. (2024), Mirzaee and Sartaj (2022)
	Boehm Titration	• Quantifies acidic surface functional groups influencing polar molecule adsorption	Oh et al. (2015), Lee and Ahmad Zaini (2022), Abdollahzadeh et al. (2022), Alam et al. (2024)
	pHpzc	• Determines point of zero charge to evaluate electrostatic interactions with pollutants	Deivayanai et al. (2024), Lee and Ahmad Zaini (2022), Wang et al. (2021)
Magnetic	VSM	• Characterize magnetic properties (saturation magnetization and superparamagnetic behavior	Abdel-Aziz et al. (2023), Abdollahzadeh et al. (2022), Ahmad et al. (2021), Alam et al. (2024), Alameri et al. (2024), Allahkarami et al. (2022), Allahkarami et al. (2023), Allwar et al. (2020), Arul et al. (2023), Barzegarzadeh and Amini-Fazl (2025b), Bouider and Rida (2024), Deivayanai et al. (2024), Farhana et al. (2022), Fitri et al. (2024), Foroutan et al. (2018), Foroutan et al. (2022), Gheitasi et al. (2022), Hammouda et al. (2022), Hashemi and Soleimani (2025), Hemalatha et al. (2024), Jain et al. (2018), Juang et al. (2018), Kakavandi et al. (2015), Kartika Santosa and Rizkiana (2023), Khaleghi et al. (2022), Khalil et al. (2024), Kord Mostafapour et al. (2024), Le et al. (2019), Lee and Ahmad Zaini (2022), Liu et al. (2022), Louis et al. (2023), Luu et al. (2022), Lv et al. (2024), Mirzaee (2022), Mirzaee and Sartaj (2022), Ngan et al. (2024), Oh et al. (2015), Ramya et al. (2022), Rashtbari et al. (2022), Rinovian et al. (2026), Saning et al. (2024), Scalera et al. (2025), Schneider et al. (2020), Spessato et al. (2020), Sudarsan et al. (2026), Thue et al. (2024), Wang et al. (2021), Wen et al. (2022), Yu et al. (2022), Zadeh and Jafari (2024), Zhang et al. (2022)
	AAS	• Measures bulk concentration of metals in the composite	Kartika Santosa and Rizkiana (2023), Louis et al. (2023), Moussa et al. (2021)
	ICP-OES	• Detects and measures trace elemental concentrations	Abdel-Aziz et al. (2023), Khaleghi et al. (2022), Spessato et al. (2020)
	ICP-MS	• High-precision elemental analysis at ultra-trace levels	Le et al. (2019), Wang et al. (2021)
​	XRD	• Identifies crystalline phases (e.g., Fe3O4), domain size, phase purity, and overall crystallinity	Abdel-Aziz et al. (2023), Abdollahzadeh et al. (2022), Alam et al. (2024), Alameri et al. (2024), Allahkarami et al. (2022), Allahkarami et al. (2023), Amili et al. (2026), Arul et al. (2023), Barzegarzadeh and Amini-Fazl (2025b), Bouider and Rida (2024), Chen et al. (2018), Farhana et al. (2022), Fitri et al. (2024), Foroutan et al. (2018), Foroutan et al. (2022), Gheitasi et al. (2022), Hammouda et al. (2022), Hardian et al. (2021), Hemalatha et al. (2024), Jain et al. (2018), Juang et al. (2018), Kakavandi et al. (2015), Kartika Santosa and Rizkiana (2023), Khaleghi et al. (2022), Khalil et al. (2024), Kord Mostafapour et al. (2024), Le et al. (2019), Lee and Ahmad Zaini (2022), Louis et al. (2023), Luu et al. (2022), Lv et al. (2024), Manousi et al. (2021), Mirzaee (2022), Mirzaee and Sartaj (2022), Moussa et al. (2021), Ngan et al. (2024), Oh et al. (2015), Ramya et al. (2022), Rashtbari et al. (2022), Rinovian et al. (2026), Saleh et al. (2017), Saning et al. (2024), Scalera et al. (2025), Schneider et al. (2020), Spessato et al. (2020), Subha et al. (2018), Sudarsan et al. (2026), Thue et al. (2024), Wan et al. (2020), Wang et al. (2021), Wen et al. (2022), Zadeh and Jafari (2024), Zhang et al. (2022)
	TEM	• Sub-nanometer visualization of particle size, shape, dispersion, lattice fringes, and core-shell structure	Allahkarami et al. (2023), Amili et al. (2026), Foroutan et al. (2018), Jain et al. (2018), Juang et al. (2018), Kakavandi et al. (2015), Khaleghi et al. (2022), Kord Mostafapour et al. (2024), Le et al. (2019), Louis et al. (2023), Luu et al. (2022), Mirzaee and Sartaj (2022), Rashtbari et al. (2022), Spessato et al. (2020), Sudarsan et al. (2026), Wang et al. (2021)
	TGA	• Evaluates thermal stability, decomposition profiles, organic content, and magnetic load percentage	Sudarsan et al. (2026), Jain et al. (2018), Seeharaj et al. (2019), Foroutan et al. (2018), Louis et al. (2023), Abdollahzadeh et al. (2022), Khaleghi et al. (2022), Spessato et al. (2020), Rashtbari et al. (2022), Kartika Santosa and Rizkiana (2023), Alam et al. (2024), Liu et al. (2022), Ramya et al. (2022)
	Contact Angle Measurement	• Measures surface wettability (hydrophobicity/hydrophilicity) and pollutant interaction	Bouider and Rida (2024), Mirzaee and Sartaj (2022)
	AFM	• 3D topography imaging of surface details, particle distribution, and micro/nano-porosity	Allwar et al. (2020)

As can be seen in Table 4, the integration of surface, magnetic and physical characterization methods can lead to a comprehensive understanding of ACMCS. For example, surface characterization techniques, including FTIR, AFM, and XPS, reveal vital details regarding topography, functional groups (such as C=O, –OH, and C-O), and surface chemistry. VSM and other magnetic characterization instruments are essential for examining the material’s magnetic characteristics. To deliver high-resolution data on the crystalline structure, morphology, particle size, surface area, and thermal stability can all be better understood using physical characterization methods as XRD, SEM, FESEM, TEM, BET, and TGA. In contrast, methods such as BET and TGA provide information on surface area, porosity, and thermal stability. Together with carbon from the activated carbon and oxygen from the magnetic material, EDX and EDS examine elemental composition. By deepening our knowledge of the properties and behavior of ACMCs composites, every technique has advantages and disadvantages, which influence the technique selection. These methods are applied in a variety of contexts, contingent on availability (materials, equipment, and expertise are accessible), cost-effectiveness (performance, cost, and scalability are balanced), and nature (material properties, functionality, and suitability for particular applications). The following succinctly describes the primary determinants that will influence the selection of characterization techniques:

1. The type of ACMCs composites and constituents, such as metals, polymers, or ceramic matrices, requires different techniques due to differences in their properties.
2. The specific property needs to be characterized strongly determines the method. These properties include mechanical, thermal, electrical, magnetic, and microstructural.
3. Scale and resolution requirements, which include ACMCs micro**/**Nano-scale.
4. Environment and testing conditions such as high or low temperature, humidity, or chemical exposure.
5. Accuracy, Sensitivity, and Repeatability: Some analyses demand sensitive and repeatable tests.
6. Cost and equipment availability: Some tests need advanced and cost-prohibitive methods. For this reason, the analysis method could depend on the lab capabilities and budget constraints.
7. Standards and Certification Requirements: Some fields often require testing methods that comply with some standards and need product certification

Finally, all of the above factors determine the analysis that could be applied to the prepared ACMCs. To provide a structured overview for researchers selecting characterization workflows, Table 5 summarizes the core analytical functions, key advantages, and operational limitations of these primary techniques.

**TABLE 5:** Advantages and limitations of key characterization techniques applied to ACMCs.

Technique	Key advantages	Major limitations/Technical challenges
FTIR	Rapid, non-destructive, cost-effective, directly identifies surface groups	Qualitative/semi-quantitative; weak signal absorption in dark, carbonaceous matrices
SEM/FESEM	High spatial resolution, visual observation of particle distribution	Localized view (risk of unrepresentative sampling); requires conductive coating
TEM/HRTEM	Ultra-high sub-nanometer resolution; verifies core-shell/encapsulation	Complex sample preparation; expensive; localized sampling area
EDX/EDS	Coupled with SEM/TEM; fast multi-element qualitative/quantitative mapping	Poor accuracy for ultra-light elements (H, C); no oxidation state data
XPS	Highly accurate elemental state and chemical bond identification	Shallow probing depth (1–10 nm); high cost; requires ultra-high vacuum
BET/N_2_ Isotherms	Standard method for textural profiling; differentiates meso/micropores	Long analysis times; assumes rigid pore model; potential micropore kinetic diffusion limitations
XRD	Identifies specific oxide phases (Fe3O4 vs. γ-Fe_2_O_3_); non-destructive	Low sensitivity to amorphous phases; carbon matrix background halos can obscure peaks
VSM	Directly quantifies magnetic separability and superparamagnetic nature	Bulk average measurement only; requires precise sample mass calibration
TGA/DTG	Accurate measurement of inorganic magnetic loading vs. carbon loss	Destructive method; overlapping decomposition regimes of complex mixtures
Raman	Sensitive to graphitic order and carbon framework defects	Fluorescence interference from raw/modified biomass matrix

Table 5 indicates that each analytical method possesses distinct advantages and limitations, technique selection depends on several practical determinants:

- Composite Matrix Type: The nature of ACMC matrix constituents (e.g., metals, polymers, carbon framework).
- Targeted Material Properties: The specific physical, chemical, thermal, magnetic, or structural parameter being targeted.
- Resolution and Scale: Resolution requirements ranging from bulk micro-scale to sub-nanometer atomic scale.
- Environmental Testing Conditions: Operational testing conditions (e.g., temperature ranges, vacuum levels, atmospheric/chemical exposure).
- Analytical Precision: Sensitivity, detection limits, and statistical repeatability required.
- Cost and Accessibility: Equipment accessibility, operational expenses, and laboratory budget constraints.
- Standardization and Compliance: Standardization protocols and regulatory testing compliance.

## 4 Applications of ACMCs

Activated carbon magnetic composites (ACMCs) represent a strategic solution to the separation bottleneck inherent in traditional water treatment. While powdered AC possesses an exceptional internal surface area and pore volume, its recovery from treated effluents is technically challenging, often requiring expensive membrane filtration or high-speed centrifugation (Allahkarami et al., 2022; Fröhlich et al., 2019). Table 6 lists the main applications of ACMCs.

**TABLE 6:** Main applications of ACMCs.

Main application	Composite	Specific application	Main findings	Ref.
Organic adsorbents	PbFe_2_O_4_ spinel-activated carbon	Removal of phenol	The material outperformed pure activated carbon in absorbing phenol from wastewater, Q_*max*_ at 25 °C = 158.94 mg/g	Allahkarami et al. (2023)
	NiFe_2_O_4_/AC composite	Removal of pharmaceuticals from diluted solutions	The material exhibited high surface area of 564.4 m^2^/g, and sufficient magnetic properties for separation by a magnetic field.	Fröhlich et al. (2019)
	Coconut shells (Cb’s) Carbonized biomass and a precursor of FeCl_3_.6H_2_O	Removal of sunset yellow (SY) food dye	Microporous ACMCs that contains magnetite, maghemite and hematite, with Q_*max*_ of 22.30 mg/g	Cazetta et al. (2016)
Inorganic adsorbents	Magnetic Fe_3_O_4_-AC(M-AC)	Removal Cr(VI) from wastewater	Q_*max*_ of ACMCs are higher than their non-magnetic counterparts. Q_*max*_ increased from 51.84 to 58.39 mg/g as the T increased from 25 °C to 40 °C, indicating an endothermic process	Al-Qodah et al. (2023)
	​	​	​	Shewatatek et al. (2025)
	MgAl-augmented double-layered hydroxide (Mg-Fe/LDH)” with sludge-based AC, (SBAC-MgFe)	Removal of phosphate and nitrate from wastewater	Q_*max*_ values were 110 and 54.5 mg/g, for phosphate and nitrate, respectively, Physisorption mainly governed the adsorption process	Alagha et al. (2020)
Catalysts	Granular AC(GAC) stabilized on Fe_3_O_4_ nanoparticles	Removal of humic acid (HA) using a catalytic ozonation process (COP)	At optimum conditions, the catalytic ozonation and single ozonation processes achieved removal efficiencies of 95% and 76.8% for HA and 87.67% and 67.1% for TOC, respectively	Zazouli et al. (2021)
	Magnetic AC(Fe_3_O_4_/AC or ACMCs)	Catalytic ozonation of the antibiotic cefixime using persulfate activated by (Fe_3_O_4_/AC or ACMCs)	Under optimal conditions (pH 8, cefixime 57.5 mg/L, ACMCS 1.5 g/L, persulfate 1.75 mM, and contact time 46.25 min), a removal efficiency of 97% was achieved	Shahbeiki et al. (2025)
	Magnetic AC(ACMCs) nanocomposite	Tetracycline (TC) degradation in aqueous solutions	Under the optimum conditions, the maximum TC removal efficiency reached 97.3% in synthetic wastewater	Asadzadeh et al. (2025)
Support for biocatalysts	A sand core coated with a magnetite layer, followed by a s layer of either fly ash or AC using epoxy resin	Immobilization of cells to be used in multi-phase bioreactors	The composite particles exhibited robust affinity for resting *E. coli* cells. Q_max_ of 18 mg/g (1.22 × 10^6^ cells) and 14 mg/g (0.95 × 10^6^ cells), respectively, with 30% higher conversion rate	Al-Qodah et al. (2015)
	Activated carbon/magnetite composite	Immobilization of the enzyme soybean peroxidase (SP) to obtain a reusable and sustainable biocatalyst	Optimal yield (100%) using a support to enzyme ratio of 1:0.05 g, a 7-h operation period at 50 °C in a 0.1 mol/L, pH 3.0. High operational stability, high performance throughout 11 consecutive reuse cycles	Torres et al. (2018)
Other applications	Carbon containing magnetic cobalt and nitrogen groups	Pre-concentration using a magnetic dispersive solid-phase extraction (MDSPE) of metal ions wastewater samples prior to inductively coupled plasma optical emission spectroscopy (ICP OES)”	Analytical sensitivity was significantly enhanced by achieving high enrichment factors, ranging from 3.2 to 13, compared to direct ICP-OES analysis	Abellán-Martín et al. (2023)
	Coal based powder magnetic AC(ACMCs)	Removal of “perfluorinated compounds (PFCs)” in treated landfill leachate effluents	Under optimized conditions the removal efficiencies were 72.8%–89.6% of perfluorohexanoic and perfluoroheptanoic acids from the leachate samples	Zhang et al. (2018)
	Iron oxide–AChybrid composites	Enhancing the microwave absorption efficiency of FeO	The IO/AC hybrid composite exhibited exceptional microwave absorption performance, achieving a maximum efficiency exceeding 99.9%	Bello et al. (2025)

Table 6 shows a summary of the main applications of ACMCs and the main findings achieved in some researches.

While ACMCs demonstrate versatility in catalysis and bio-immobilization, the clear majority of their applications remain centered on environmental adsorption of pollutants. Consequently, the modelling and optimization sections focuses exclusively on the mathematical, kinetic, and isotherm modeling of these adsorption systems.

By embedding magnetic nanoparticles, such as nickel ferrite (NiFe_2_O_4_), lead ferrite (PbFe_12_O_19_), or magnetite (Fe_3_O_4_), into the carbonaceous matrix, researchers have created smart adsorbents. These materials can be manipulated via an external magnetic field, achieving nearly 100% recovery from aqueous phases (Allahkarami et al., 2022). This hybrid approach not only preserves the high surface reactivity of the carbon but also adds a layer of practical handling, making these composites suitable for large-scale industrial applications and urban drinking water treatment plants (Fröhlich et al., 2019). Applications extend beyond simple adsorption to include specialized roles in microwave absorption or electromagnetic wave shielding, specialized chemical synthesis, and advanced catalysis (Hemalatha et al., 2024; Mirshafiee et al., 2022; Zhao et al., 2019).

### 4.1 ACMCs as adsorbents

Adsorption remains the most versatile and cost-effective strategy for environmental remediation, particularly when utilizing magnetic carbon frameworks that bypass the need for traditional centrifugation or filtration (Niculescu et al., 2024). Research indicates that the impregnation of magnetic nanoparticles, such as nickel ferrite (NiFe_2_O_4_) or iron salts, into the carbon phase does not result in a continuous, pore-blocking coating. Instead, these particles are distributed as a discontinuous phase, which helps address the tendency of Nano-sized ferrites to agglomerate (Moussa et al., 2021). This architectural arrangement potentially augments the porosity of the material and maintains a large specific surface area, with some catalysts exhibiting a shell-like structure and abundant adsorption groups. Such a configuration facilitates rapid mass transfer, leading to high kinetic rates where sorption processes often follow pseudo-second-order reaction models. Consequently, these magnetic composites demonstrate a retention ability towards pollutants like (Cu(II), Zn(II)) ions and organic dyes that is significantly superior to that of pristine activated carbon, while allowing for easy and fast recovery via an external magnetic field (Moosavi et al., 2020; Moussa et al., 2021; Yu et al., 2022). Furthermore, the metal salts, such as FeCl_3_ or ZnCl_2_, often function as catalytic activating agents during thermal treatment, effectively etching the carbon surface to develop a more extensive hierarchical pore network (Lee and Ahmad Zaini, 2022). This augmented porosity and discontinuous particle distribution directly facilitate rapid mass transfer by providing a higher density of open diffusion channels, allowing pollutants to penetrate deep into the material’s interior through diminished intraparticle diffusion resistance. Consequently, this architecture drives high kinetic rates, ensuring rapid attainment of adsorption equilibrium. The presence of the magnetic core ensures that this highly porous system can be efficiently separated from aqueous media via an external magnetic field, maintaining structural integrity and high recyclability without compromising the primary adsorption capacity of the carbon matrix (Allahkarami et al., 2022; Fröhlich et al., 2019). In addition, the structural integrity of these composites allows them to withstand various chemical environments, maintaining high adsorption capacities across a wide range of pH and temperature conditions (Saadi et al., 2024; Yanti et al., 2023). Furthermore, the robust architecture of these materials ensures they remain stable during repeated regeneration cycles, preventing the leaching of the magnetic phase into the treated effluent (Lima et al., 2025; Meng et al., 2025).

#### 4.1.1 ACMCs as organic adsorbents

ACMCs exhibit a diverse range of interaction mechanisms for organic pollutants, including hydrogen bonding, π-π electron interactions, and hydrophobic effects. For instance, Figure 4 shows the interaction mechanisms between the surface of the prepared magnetic ACcomposite and a dye molecule.

**FIGURE 4:**
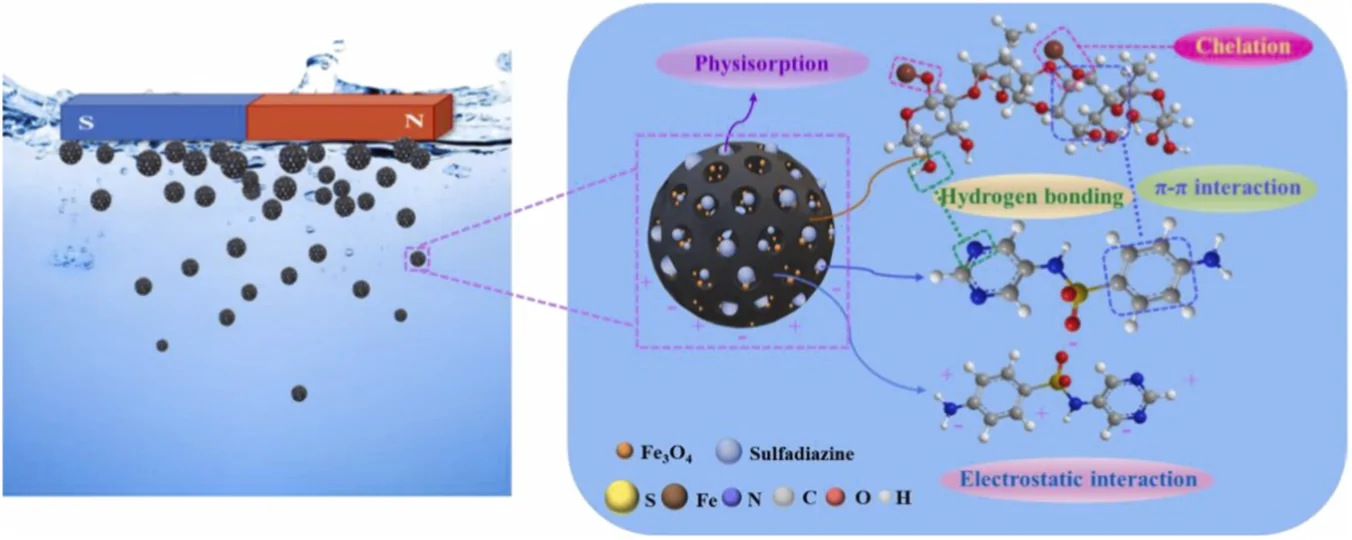
A schematic representation of the adsorption mechanism of sunset yellow food dye onto the synthesized magnetic activated carbon composite (ACMCs) (Yang et al., 2023).

The following list shows examples of the organic pollutants that can be separated via adsorption onto ACMCs.

- Pharmaceuticals: In studies involving emerging contaminants, a hydrothermal NiFe_2_O_4_/AC composite (NiAC) showed high affinity for non-steroidal anti-inflammatory drugs. It achieved a maximum adsorption capacity (Q_*max*_) of 261.35 mg/g for ibuprofen and 97.75 mg/g for ketoprofen (Fröhlich et al., 2019). The process was identified as spontaneous and endothermic, with the removal efficiency heavily influenced by the solution pH. Another study, used a “single-step pyrolysis” to produce magnetic ACfrom “tucumã” (“Astrocaryum aculeatum”) seed with NiCl_2_ and ZnCl_2_ (Thue et al., 2020). The resulting material (MT-2.0) was used for the treatment of synthetic pharmaceutical wastewater, removing up to 99.1% of organic compounds with Q_*max*_ of 199.3 and 335.4 mg/g for nicotinamide and, propranolol respectively.
- Phenolic Compounds: Phenol removal is a critical target due to its high toxicity. A novel “lead ferrite-ACMCs” composite achieved a Q_*max*_ of 145.708 mg/g for phenol, significantly outperforming pristine AC (Allahkarami et al., 2022). The enhancement is referred to the metal hydroxides present on the AC surface, which facilitate additional electrostatic interactions with the phenol. Another study detailed the synthesis of a “magnetic cobalt activated carbon (MCAC)” composite designed for the rapid sequestration of phenol (Mohammadi et al., 2020). By impregnating cobalt nanoparticles as a discontinuous phase onto a porous carbon framework, the researchers developed an adsorbent that facilitates high mass transfer rates and achieves a Q_*max*_ of 107.5 mg/g. The process was shown to follow Elovich kinetic and Langmuir isotherm models, indicating a chemisorption mechanism.
- Industrial Dyes: The composites have been successfully tested against various dyes (e.g., methylene blue and Congo red), proving that the magnetic phase does not block the essential micropores of the carbon, thus maintaining high kinetic rates. For instance, a study demonstrated that the synthesis of ACMCs via a discontinuous distribution of magnetic nanoparticles prevented the occlusion of the carbon framework’s micropores (de Souza Luz Faria et al., 2026). This architectural strategy yields a highly accessible hierarchical pore network, resulting in a (Q_*max*_) of 142.8 mg/g for MB. The adsorption kinetics followed pseudo-second-order model, and rapidly reached equilibrium due to enhanced mass transfer. A second study explored the synthesis of a sustainable orange peel-derived magnetic activated carbon (OP-ACMCs) for the efficient sequestration of Acid Orange 7 (AO7) dye (Khalil et al., 2024). By utilizing agricultural waste and incorporating a magnetic phase, the researchers developed an adsorbent that maintains a high specific surface area and a porous architecture, facilitating rapid mass transfer of the anionic dye molecules. The adsorption process reached a maximum capacity (Qmax) of 120.48 mg/g, with a good fit with pseudo-second-order kinetic and Langmuir isotherm models.

#### 4.1.2 ACMCs as inorganic adsorbents

The removal of inorganic species, primarily toxic heavy metals and oxyanions, relies on the formation of complexes and ion exchange at the functional groups (carboxyl, hydroxyl) of the carbon framework. These interactions are enhanced by the structural synergy between the carbonaceous matrix and the inorganic magnetic phase. The magnetic core does more than just aid recovery; it significantly influences the surface charge of the composite. By adjusting the metal-to-carbon ratio during synthesis, the point of zero charge (pHpzc) can be strategically shifted to optimize the electrostatic attraction of divalent metal cations or anionic species from complex wastewater matrices (Liu et al., 2022) as shown in Figure 5.

**FIGURE 5:**
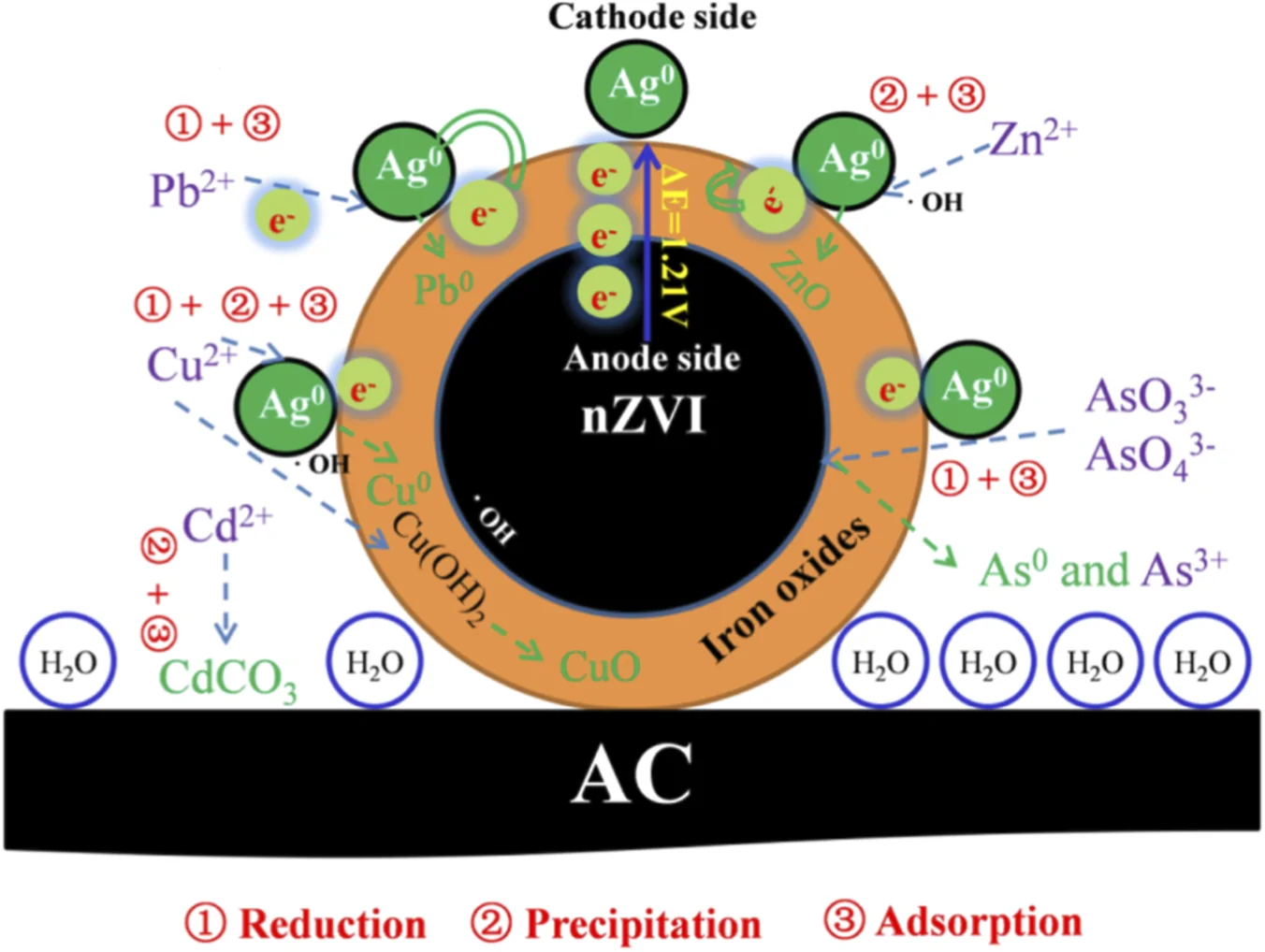
Interfacial reaction mechanism of cationic heavy metal and arsenic removal by nAg/nZVI/AC (Liu et al., 2022).

For instance, Figure 5 shows the interactions mechanisms between carbon-based and metal ions. ACMCs composites has been tested for the following inorganic pollutants:

- Heavy Metals: Research has extensively focused on the sequestration of Pb(II), Cd(II), and Cr(VI). Magnetic AC composites facilitate the removal of these ions through monolayer adsorption on a homogeneous surface, where the pollutants occupy specific active sites until saturation is reached (Arul et al., 2023). Studies have shown that utilizing low-cost precursors, such as tire waste, can achieve up to 99.5% removal efficiency for Cr(VI), even in complex industrial matrices like tannery wastewater (Ahmad et al., 2021). A second study synthesized a nickel ferrite (NiFe_2_O_4_) magnetic ACcomposite specifically for sequestering Pb^2+^ and Cd^2+^ ions (Moussa et al., 2021). The material achieved a (Q_*max*_) of 105.3 mg/g and 34.8 mg/g, for Pb^2+^ and Cd^2+^ ions, respectively. The adsorption mechanism followed the Langmuir isotherm, implying the formation of a monolayer coverage on the surface. For the treatment of hexavalent chromium, the composite’s architecture facilitates a critical dual-action mechanism: the carbon matrix serves as a reducing agent that converts toxic, mobile Cr(VI) into the less toxic and less mobile Cr(III) state. This reduced species then precipitates onto or is complexed by the magnetic nanoparticles within the framework (Guo et al., 2021; Mohamed et al., 2024). Recent studies have demonstrated that even low-cost ACMCs derived from fly ash and sand can achieve significant adsorption capacities, approximately 50 mg/g, for these toxic chromium ions (Al-Qodah et al., 2023).
- Beyond simple cations, magnetic AC(ACMCs) composites are highly effective against toxic metalloids, specifically arsenic (As(III)/As(V)) and antimony (Sb). The magnetic iron oxide core, often composed of magnetite or maghemite, provides a natural high affinity for arsenic species through the formation of stable inner-sphere complexes via ligand exchange with surface hydroxyl groups (Liu et al., 2015; Müller et al., 2010).
- Nutrients and anions: Magnetic hybrids are increasingly applied to the removal of phosphates (PO_4_^3-^), nitrates (NO_3_^−^) and fluoride (F^−^). For instance, in a study, a nano-magnetically modified ACusing oak shells was developed as a sustainable adsorbent for fluoride ions removal from wastewater. It confirmed that the adsorption followed the Freundlich isotherm, indicating multilayer coverage on a heterogeneous surface, while the (Q_*max*_) predicted was 454.54 mg/g (Takmil et al., 2020). In addition, by doping the carbon with magnesium or aluminum, researchers have created specialized sites for selective ion exchange to prevent environmental issues like eutrophication and health concerns like fluorosis (Alagha et al., 2020; Dar and Kurella, 2025; Khalil et al., 2022).
- Radionuclides: In specialized applications, ACMCs are used to trap isotopes such as cesium (Cs^137^) and strontium (Sr^90^). The magnetic properties are critical here, as they allow for remote, automated recovery of the adsorbent from hazardous nuclear wastewater without direct human contact. For instance, Alagha, et al. (2020), investigated the synthesis, characterization, and testing of a unique, magnetically separable, and eco-friendly adsorbent composite material for the advanced treatment and efficient removal of nitrate and phosphate pollutants from wastewater. The maximum adsorption capacities of phosphate and nitrate were 110 and 54.5 mg/g, respectively. The competition between phosphate and coexisting ions such as Cl^−^, CO_3_^2−^, and SO_4_^2−^ was studied and found to be remarkably lower in comparison with the nitrate adsorption.

Table 7 shows that different types of ACMCs applied as adsorbents for organic and inorganic pollutants. In all applications, ACMCs proves that it has a large surface area, high adsorption capacity and high removal efficiencies. These results encourage the use of these ACMCs as selective high capacity adsorbents for different types and concentration pollutants.

**TABLE 7:** Summarizes some published results concerning ACMCs use for the adsorption of organic and inorganic pollutants.

Ref.	ACMC	Surface area m^2^/g	q_max_ (mg/g)	*R* ^*2*^	Removal efficiency %	Pollutant
Moussa et al. (2021)	AC- NiFe_2_O_4_	564.4	261.35 and 97.75	​	90	ibuprofen and ketoprofen
Zadeh and Jafari (2024)	AC-alginate/Fe_3_O_4_	​	37.764	>0.99	97	Pb^+2^
Kakavandi et al. (2015)	(Fe_3_O_4_@C)	​	71.42	​	90	Pb^+2^
Thue et al. (2020)	AC-NiCl_2_	1281	199.3 and 335.4	​	99.1	Nicotinamide, propanolol
Yang et al. (2008)	BPAC@Fe_3_O_4,_ SSAC@Fe_3_O_4_	394.85 and 160.02/cm^2^/g	333.33 and 303.03	0.9886	​	MB or Congo Red
Barzegarzadeh and Amini-Fazl (2025a)	MnFe_2_O_4_	43.86	152	0.986	99.95	Abamectin
Yao et al. (2014)	AC-Iron oxide	678.3	20.24	0.9996	95.27	As(V)
Altıntıg et al. (2017)	Fe-AC	​	357.1	​	95	MB
Hardian et al. (2021)	AC-MgFe_2_O_4_	88.134	51.4	0.96	99.26	MB
Saleh et al. (2017)	polyethyleneimine AC/Fe	​	115.31	0.978	​	U(VI)
Foroutan et al. (2018)	AC-CoFe_2_O_4_	​	70.422	0.9999	0.9435	Cr(VI)
Abdollahzadeh et al. (2022)	AC-Fe_3_O_4_	421.11	119.05	0.9541	93.22	Reactive blue 19
Allahkarami et al. (2022)	Lead ferrite-AC	747.53	145.708	​	99.17	Phenol
Spessato et al. (2020)	MGAC	​	20.2	0.96	99.3	PAHs
Alameri et al. (2024)	AC-Fe_3_O_4_	1401	178.8	0.985	100	Ciprofloxacin
Alam et al. (2024)	AC-Iron oxide	​	454.545	0.955	95	Acid orange 8
Ngan et al. (2024)	AC-FeCl_2_	417.13	62.885	0.885	​	Cr(VI)
Gheitasi et al. (2022)	747.71	109.37	107.5	0.99	​	mercury (II)
Saadi et al. (2024)	AC-FeCl_3_	264.97	198.529	0.938	92.28	Rhodamine B
Zhang et al. (2021)	AC-FeCl_3_	1436	253.2 and 73.3	0.879	​	Pb(II) and Cd(II
Yağmur and Kaya (2021)	AC-FeCl_3_	747.71	156.25	0.9157	88	MB
Ahmed et al. (2026)	Fe-modified PAC	28.7	​	​	67	Ciprofloxacin
Romero et al. (2026)	AC-FeCl_3_	1175	714	0.99	100	pharmaceuticals

### 4.2 ACMCs as catalysts

ACMCs are increasingly utilized as heterogeneous catalysts in advanced oxidation processes (AOPs). Because they possess both conductive carbon frameworks and redox-active magnetic cores (like spinals), they serve as potent activators for oxidants such as hydrogen peroxide or persulfate. Figure 6 shows tetracycline (TC) removal efficiency with various processes.

**FIGURE 6:**
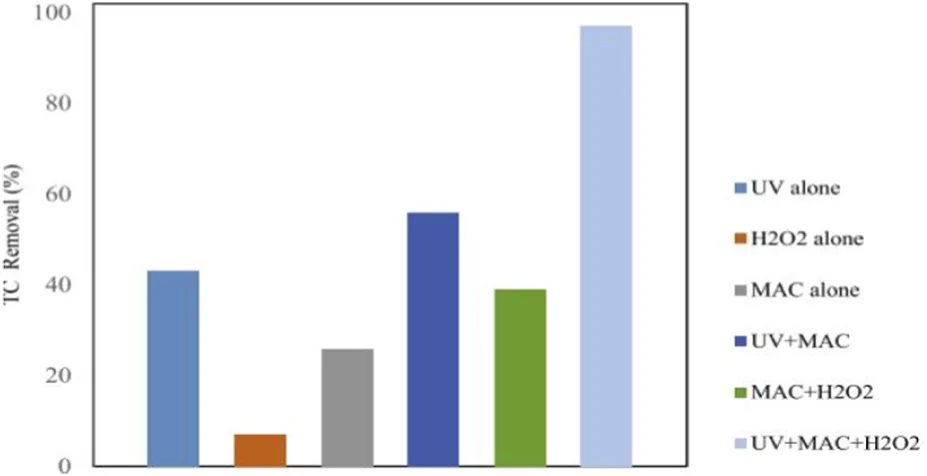
Tetracycline (TC) removal efficiency with various processes (pH = 4, concentration of TC = 5 μM, concentration of H_2_O_2_ = 3 mM, dosage of ACMCs = 1.2 g/L and T = 25 °C). Reprinted with permission from (Asadzadeh et al., 2025).

Figure 6 shows the enhanced performance of ACMCs when used with H_2_O_2_ and UV radiation. The iron or nickel species within the composite can facilitate electron transfer, leading to the generation of highly reactive hydroxyl (^**.**^OH) and sulfate (SO4^**.**−^) radicals that mineralize recalcitrant organic pollutants. For instance, a study investigated the catalytic ozonation process (COP) using magnetic AC(ACMCs) as a heterogeneous catalyst to enhance the mineralization of humic acid (HA) from aqueous solutions. By serving as a mediator for electron transfer, the ACMCs facilitated the decomposition of molecular ozone into highly reactive hydroxyl radicals (^**.**^OH), shifting the reaction toward a more potent advanced oxidation pathway. This mechanism allowed for significantly higher total organic carbon (TOC) removal and mineralization compared to ozonation alone, effectively preventing the formation of harmful by-products (Zazouli et al., 2021). Another study investigated an integrated persulfate (PS) and ozonation (O_3_) process catalyzed by an Fe_3_O_4_/AC composite for the degradation of the antibiotic cefixime (Shahbeiki et al., 2025). By utilizing the magnetic ACas a dual-activator, the system created a synergistic radical cocktail of hydroxyl (^**.**^OH) and sulfate (SO4^**.**−^) radicals, which significantly enhanced mineralization compared to individual oxidation methods.

### 4.3 Composite magnetic activated carbon biocatalyst supports

The hierarchically porous structure of ACMCs, characterized by a complex network of cavities and protuberances, provides an ideal scaffold for the immobilization of biological agents through various mechanisms such as covalent bonding and physical adsorption as shown in Figure 7.

**FIGURE 7:**
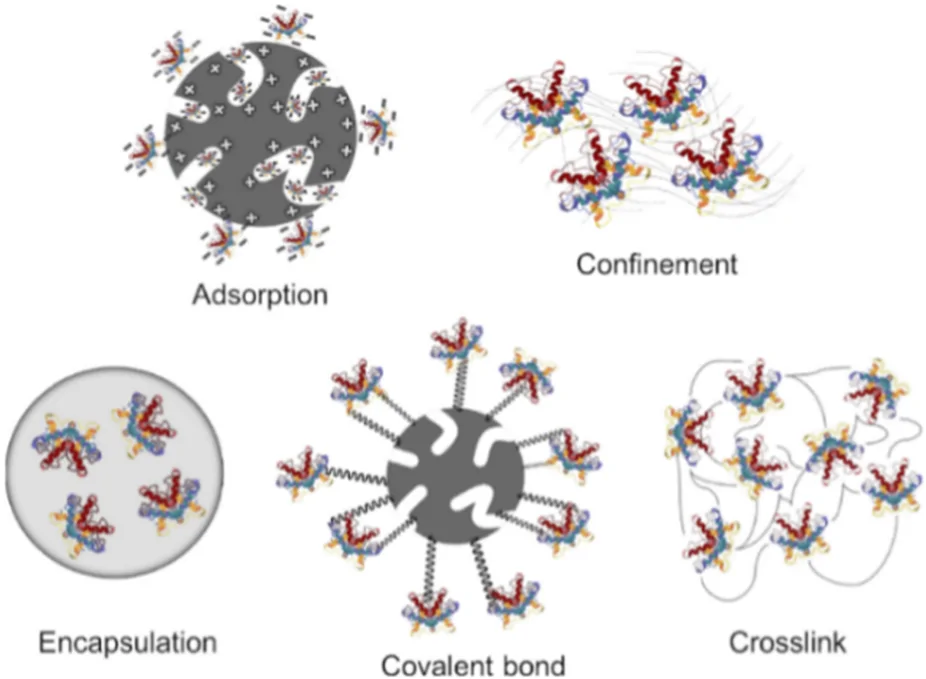
A diagram illustrating the methods of enzyme immobilization to a support material. Reprinted with permission from (Santos et al., 2024).

The carbon matrix serves as a protective buffer, shielding sensitive microbial cells or enzymes, such as laccase, from inhibitory environmental fluctuations in temperature and pH (Nooin et al., 2026; Torres et al., 2018; Wang et al., 2024). In addition, biological modification of ACMCs often involves the use of functional linkers to stabilize enzymes. Furthermore, the integration of magnetic phases like NiFe_2_O_4_ or PbFe_12_O_19_ addresses the primary economic hurdle in biocatalytic reactors, the high cost of enzymes, by enabling the remote-controlled recovery and reuse of these biological components via external magnetic fields, significantly enhancing the process’s overall economic feasibility (Matveeva and Bronstein, 2021; Saire-Saire et al., 2020).

### 4.4 Other applications of ACMCs

Beyond standard adsorption, ACMCs have found diverse applications in advanced chemical and physical processes. They serve as high-performance agents in magnetic solid-phase extraction (MSPE) to pre-concentrate trace pollutants before analytical detection (Abellán-Martín et al., 2023; Rodrigues et al., 2020; Rodríguez et al., 2015). In environmental remediation, they are effectively applied to the treatment of complex matrices like landfill leachate and the removal of persistent volatile organic compounds (VOCs), such as chloroform and chlorobenzene (Reshadi et al., 2020). In these contexts, the carbon matrix provides the necessary adsorption capacity while the magnetic component facilitates the separation of the adsorbent from complex industrial effluents. For example, a study evaluated the use of coal-based magnetic AC(ACMCs) to remove persistent perfluorinated compounds (PFCs) from complex landfill leachate effluents (Zhang et al., 2018). The ACMCs achieved high removal efficiencies (up to 89.6%) by leveraging its hierarchical porosity to trap diverse PFC chain lengths. Furthermore, emerging research highlights their potential in microwave absorption; the synergy between the dielectric loss of the carbon framework and the magnetic loss of the ferrite phase makes these composites excellent candidates for electromagnetic interference (EMI) shielding (Figure 8) and microwave-induced catalytic degradation (Bello et al., 2025; Lataf et al., 2024; Zhao et al., 2019).

**FIGURE 8:**
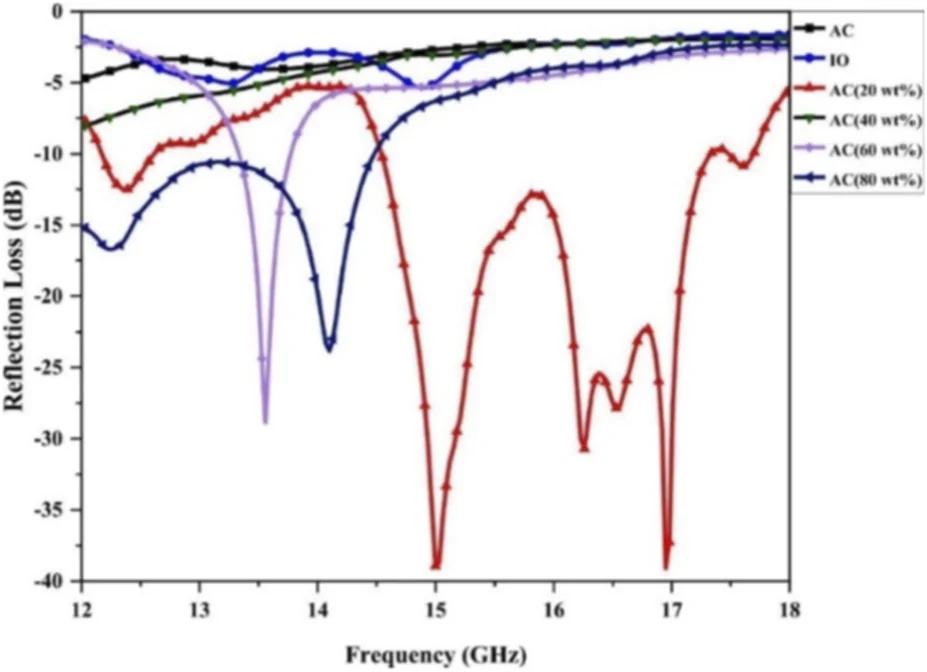
Reflection loss curves for iron oxide/activated carbon (IO/AC) hybrid composites with different IO contents (w %). Reprinted with permission from (Bello et al., 2025).

## 5 Modeling of ACMCs processes

Studies that investigated magnetic AC composites have used three main types of mathematical models, which can be summarized as follows:

1. Models used to understand process mechanisms and kinetics.
2. Models used to optimize the synthesis or operational parameters in order to improve the performance efficiencies and reduce the operating in addition to the capital cost.
3. Other models used to describe processes occurring at the surface of ACMCs.

Consequently, the subsequent subsections evaluate the specific mathematical models applied to various processes involving magnetic ACcomposites.

### 5.1 Kinetic and isotherm modelling of ACMCs adsorption processes

The majority of the studies have used ACMCs in adsorption applications. Thus, kinetic and equilibrium isotherm studies have been performed in order to fit the experimental data and understand the mechanisms of the sorption process onto ACMCs. The following two tables summarize the kinetic and isotherm models that have been used in the literature for ACMCs. The kinetic models are used to understand the dynamics of the adsorption process and explain the possible mechanisms for the interactions and the diffusion style of the adsorbate molecules or ions towards the surface of ACMCs. Adsorption can occur in two general modes: chemical or physical adsorption. In the physical mode, the target molecules are attached to the surface of adsorbent pore walls by weak forces, and the heat of adsorption is low, only slightly greater than the heat of sublimation of the adsorbent. In the chemical adsorption process, the target adsorbate makes covalent bonds to specific sites on the adsorbent, with a much higher heat of adsorption, approximately equal to the heat of reaction (Berger and Bhown, 2011). The values of the heat of adsorption for the physio and chemisorption processes lies in the range of −25 to −40 −60 to −100 kJ/mol, respectively (Gunawardene et al., 2022).

Table 8 lists the kinetic models applied in the literature to study the adsorption kinetics of ACMCs. These models include the Lagergren pseudo-first-order kinetic model (Lagergren, 1898), the Ho’s pseudo-second-order kinetic model (Ho and McKay, 1998), Elovich model (Elovich, 1962), Michaelis–Menten model, Avrami (Avrami, 1939), (Avrami, 1940), and (Avrami, 1941) fractional-order model.

**TABLE 8:** The kinetic models applied for activated carbon magnetic composites (*ACMCs)* in the literature since the year 2000.

Kinetic model used	Model equation	Ref.	Total Ref.
The Lagergren pseudo-first-order model	$q_{t}=q_{e}\left(1-e^{-k_{1}t}\right)$	Abdel-Aziz et al. (2023), Allahkarami et al. (2022), Cazetta et al. (2016), Foroutan et al. (2022), Fröhlich et al. (2019), Khaleghi et al. (2022), Lee and Ahmad Zaini (2022), Luu et al. (2022), Shao et al. (2012), Thue et al. (2020), Tsoutsa et al. (2023), Zhao et al. (2023)	12
	$\ln\left(q_{e}{-q}_{t}\right)=\ln{\;q}_{e}-k_{1}t$	Moussa et al. (2021), Zadeh and Jafari (2024), Kakavandi et al. (2015), Saleh et al. (2017), Chen et al. (2018), Juang et al. (2018), Le et al. (2019), Foroutan et al. (2018), Wang et al. (2021), Abdollahzadeh et al. (2022), Wen et al. (2022), Bouider and Rida (2024), Saning et al. (2024), Ahmad et al. (2021), Zhang et al. (2022), Liu et al. (2022), Ramya et al. (2022), Zhang et al. (2021), Sharififard (2026), Jiang et al. (2022)	20
	$\log_{10}\left(q_{e}-q_{t}\right)={\;\log}_{10}\;q_{e}-\frac{k_{1}}{2.303}t$	Alameri et al. (2024), Arul et al. (2023), Gheitasi et al. (2022), Hardian et al. (2021), Jain et al. (2018), Li et al. (2026), Liu et al. (2023), Louis et al. (2023), Rashtbari et al. (2022), Tran et al. (2021), Yağmur and Kaya (2021)	11
The Ho’s pseudo-second-order model	$q_{t}=\frac{k_{2}{q_{e}}^{2}\;t}{\left(1+k_{2}q_{e}t\right)}$	Abdel-Aziz et al. (2023), Cazetta et al. (2016), Foroutan et al. (2022), Fröhlich et al. (2019), Khaleghi et al. (2022), Lee and Ahmad Zaini (2022), Luu et al. (2022), Shao et al. (2012), Thue et al. (2020), Tsoutsa et al. (2023), Zhao et al. (2023)	11
	$\frac{t}{q_{t}}=\frac{1}{k{q_{e}}^{2}}+\frac{1}{q_{e}}t$	Moussa et al. (2021), Zadeh and Jafari (2024), Kakavandi et al. (2015), Al-Qodah et al. (2023), Luo and Zhang (2009), Yao et al. (2014), Hardian et al. (2021), Saleh et al. (2017), Jain et al. (2018), Chen et al. (2018), Juang et al. (2018), Le et al. (2019), Foroutan et al. (2018), Wang et al. (2021), Louis et al. (2023), Abdollahzadeh et al. (2022), Wen et al. (2022), Rashtbari et al. (2022), Bouider and Rida (2024), Alameri et al. (2024), Saning et al. (2024), Ahmad et al. (2021), Zhang et al. (2022), Gheitasi et al. (2022), Liu et al. (2022), Ramya et al. (2022), Arul et al. (2023), Liu et al. (2023), Zhang et al. (2021), Sharififard (2026), Jiang et al. (2022), Li et al. (2026), Tran et al. (2021), Yağmur and Kaya (2021), Kodandoor et al. (2026)	34
	$q_{t}=\frac{t}{\left(\frac{1}{k_{2}{q_{e}}^{2}}\right)+\left(\frac{t}{q_{e}}\right)}$	Allahkarami et al. (2022)	​
Michaelis−Menten equation	$v_{o}=\frac{v_{\max}\left[S\right]}{K_{m}+\left[S\right]}$	Zheng et al. (2012)	1
Avrami fractional-order	$F=\frac{V-V_{0}}{V_{m}-V_{0}}=1-e^{-kt^{n_{\text{AV}}}}$	Thue et al. (2020)	1
Elovich model	$q_{t}=\beta \;\ln\left(\alpha \beta \right)+\beta \;\ln\;t$	Kakavandi et al. (2015)	1
	$q_{t}=\frac{1}{\beta }\ln\left(\alpha \beta t\right)$	Al-Qodah et al. (2023), Alameri et al. (2024), Foroutan et al. (2022), Li et al. (2026)	3
	$q_{t}=\frac{1}{a}\ln\left(1+abt\right)$	Allahkarami et al. (2022), Cazetta et al. (2016)	2
The Weber-Morris Intraparticle diffusion	$q_{t}=k_{i}t^{0.5}+C_{i}$	Abdel-Aziz et al. (2023), Ahmad et al. (2021), Al-Qodah et al. (2023), Bouider and Rida (2024), Foroutan et al. (2022), Jain et al. (2018), Jiang et al. (2022), Le et al. (2019), Lee and Ahmad Zaini (2022), Liu et al. (2023), Louis et al. (2023), Luu et al. (2022), Ramya et al. (2022), Rashtbari et al. (2022), Saleh et al. (2017), Saning et al. (2024), Tran et al. (2021), Yağmur and Kaya (2021), Zhao et al. (2023)	19
Boyd’s models	$B_{t}=-0.4985-\ln\left(1-F\right)=-0.4985-\ln\left(1-q_{t}/q_{e}\right)$	Alameri et al. (2024), Ramya et al. (2022)	2
	$B_{t}=-0.4977-\ln\left(1-F\right)\text{ for }F\;>\;0.85$ $B_{t}={\left(\sqrt{\pi }-\sqrt{\pi -\left(\frac{\pi^{2}F}{3}\right)}\right)}^{2}\text{ for }F\;<\;0.85$	Alameri et al. (2024), Lee and Ahmad Zaini (2022)	2
The linear driving force model (LDF)	$\frac{d\bar{q}}{dt}=k_{\text{LDF}}\left(q_{m}\frac{K_{L}\left(C_{0}-\left(m/V\right)\bar{q}\right)}{1+K_{L}\left(C_{0}-\left(m/V\right)\bar{q}\right)}-\bar{q}\right)=k_{\text{LDF}}\left(q^{*}-\bar{q}\right)$	Allahkarami et al. (2022)	1
The fractional power equation	$\log\left(q_{t}\right)=\log\left(K\right)+V\;\log\left(t\right)$	Alameri et al. (2024)	1

It is evident from Table 8 that the Lagergren pseudo-first-order model, the Ho’s pseudo-second-order model and the Weber-Morris Intraparticle diffusion model have wide applications for describing adsorption systems associated with a chemical nature (Lee and Ahmad Zaini, 2022; Zadeh and Jafari, 2024; Kakavandi et al., 2015; Zarei and Sinapour, 2015; Furlan et al., 2017; Thue et al., 2020; Niculescu et al., 2024; Al-Qodah and Al-Shannag, 2006; Sharma et al., 2022; Azam et al., 2022; Al-Qodah et al., 2015; Krishna et al., 2017; Al-Qodah et al., 2023). The Avrami kinetic model was modified from the thermal decomposition kinetics of phase changes to an adsorption-based model (Thue et al., 2020).

In addition to the aforementioned reaction kinetics models, diffusion-based models such as the Weber–Morris model (Zhang et al., 2021) and the Boyd kinetic model (Sharififard, 2026). These models are commonly applied to elucidate mass transfer mechanisms and identify the rate-controlling step. According to the Weber–Morris model, if a plot of *q*_t_ versus *t* ^1/2^ yields a straight line passing through the origin, intraparticle (pore) diffusion is the sole rate-limiting step. Conversely, a non-zero intercept indicates a degree of boundary layer control, demonstrating that the adsorption kinetics are simultaneously governed by both film and intraparticle diffusion. Similarly, in Boyd’s model, pore diffusion is identified as the rate-limiting step if the plot of *B*_t_ versus *t* is linear and passes through the origin; otherwise, film diffusion controls the adsorption rate (Jiang et al., 2022). As summarized in Table 1, the pseudo-second-order and pseudo-first-order models remain the most widely applied kinetic frameworks for ACMCs.

Table 9 shows the isotherm models applied in the literature to study the adsorption equilibrium isotherms of ACMCs. Adsorption isotherms are used to indicate the adsorbates affinity for the adsorbent.

**TABLE 9:** The isotherm models applied for activated carbon magnetic composites (ACMCs) in the literature since the year 2000.

Isotherm model	Equation	Ref.	Nu. Ref.	Total
Langmuir Isotherm	$q_{e}=\frac{q_{m}K_{L}C_{e}}{1+K_{L}C_{e}}$	Abdel-Aziz et al. (2023), Al-Qodah et al. (2023), Allahkarami et al. (2022), Allahkarami et al. (2023), Altamer et al. (2024), Altıntıg et al. (2017), Bouider and Rida (2024), Cazetta et al. (2016), Foroutan et al. (2022), Fröhlich et al. (2019), Gheitasi et al. (2022), Juang et al. (2018), Khaleghi et al. (2022), Khalil et al. (2022), Le et al. (2019), Lee and Ahmad Zaini (2022), Liu et al. (2023), Luu et al. (2022), Ramya et al. (2022), Saleh et al. (2017), Shao et al. (2012), Singh and Beg (2013), Tsoutsa et al. (2023)	23	47
	$\frac{1}{q_{e}}=\frac{1}{K_{L}q_{m}}\frac{1}{C_{e}}+\frac{1}{q_{m}}$	Al-Qodah et al. (2015), Alameri et al. (2024), Farhana et al. (2022), Hardian et al. (2021), Luo and Zhang (2009), Yang et al. (2008)	6	
	$\frac{C_{e}}{q_{e}}=\frac{1}{K_{L}q_{m}}+\frac{C_{e}}{q_{m}}$	Zadeh and Jafari (2024), Yao et al. (2014), Jain et al. (2018), Foroutan et al. (2018), Wang et al. (2021), Louis et al. (2023), Abdollahzadeh et al. (2022), Wen et al. (2022), Rashtbari et al. (2022), Saning et al. (2024), Ahmad et al. (2021), Zhang et al. (2022), Liu et al. (2022), Arul et al. (2023), Rajula et al. (2020), Li et al. (2026), Yağmur and Kaya (2021), Kodandoor et al. (2026)	17	
	$\frac{C_{e}}{q_{e}}=C_{e}\left(\frac{a_{L}}{K_{L}}\right)+\left(\frac{1}{K_{L}}\right)$	Zarei and Sinapour (2015)	1	
Freundlich Isotherm	$q_{e}=K_{F}C_{e}^{1/n}$	Abdel-Aziz et al. (2023), Ahmed et al. (2026), Al-Qodah et al. (2023), Allahkarami et al. (2022), Allahkarami et al. (2023), Altamer et al. (2024), Bouider and Rida (2024), Cazetta et al. (2016), Foroutan et al. (2022), Fröhlich et al. (2019), Gheitasi et al. (2022), Juang et al. (2018), Khaleghi et al. (2022), Khalil et al. (2022), Kodandoor et al. (2026), Le et al. (2019), Lee and Ahmad Zaini (2022), Liu et al. (2023), Luu et al. (2022), Ramya et al. (2022), Rashtbari et al. (2022), Shao et al. (2012), Tsoutsa et al. (2023), Zhao et al. (2023)	22	46
	$\ln{\;q}_{e}=\frac{1}{n}\ln{\;C}_{e}+\ln\;K_{F}$	Zadeh and Jafari (2024), Zarei and Sinapour (2015), Luo and Zhang (2009), Foroutan et al. (2018), Wang et al. (2021), Louis et al. (2023), Abdollahzadeh et al. (2022), Wen et al. (2022), Saning et al. (2024), Zhang et al. (2022), Farhana et al. (2022)	11	
	$\log{\;q}_{e}=\left(\frac{1}{n}\right)\log{\;C}_{e}+\log{\;K}_{F}$	Ahmad et al. (2021), Alameri et al. (2024), Altıntıg et al. (2017), Arul et al. (2023), Hardian et al. (2021), Jain et al. (2018), Li et al. (2026), Liu et al. (2022), Saleh et al. (2017), Shao et al. (2012), Sharififard (2026), Yao et al. (2014), Yağmur and Kaya (2021)	13	
Temkin Isotherm	$q_{e}=\beta_{T}\;\ln{\;K}_{T}+\beta_{T}\;\ln\;C_{e}$ $\beta_{T}=RT/b_{T}$	Abdel-Aziz et al. (2023), Al-Qodah et al. (2023), Alameri et al. (2024), Allahkarami et al. (2022), Allahkarami et al. (2023), Altamer et al. (2024), Arul et al. (2023), Farhana et al. (2022), Foroutan et al. (2022), Gheitasi et al. (2022), Le et al. (2019), Louis et al. (2023), Ramya et al. (2022)	13	13
Dubinin–Radushkevich (D-R) Isotherm	$q_{e}=q_{m}\;\exp\left(-\beta_{\text{DR}}{\;\varepsilon }^{2}\right)$ $\varepsilon =RT\;\ln\left(1+\frac{1}{C_{e}}\right)$ $E=\frac{1}{\sqrt{2\;K_{\text{DR}}}}$	Abdel-Aziz et al. (2023), Foroutan et al. (2022), Khaleghi et al. (2022), Lee and Ahmad Zaini (2022)	4	6
	$\ln{\;q}_{e}=\ln{\;q}_{m}-\beta_{\text{DR}}\varepsilon^{2}$ $\varepsilon =RT\;\ln\left(1+\frac{1}{C_{e}}\right)$ $E=\frac{1}{\sqrt{2\beta_{\text{DR}}}}$	Alameri et al. (2024), Foroutan et al. (2018)	2	
The Redlich–Peterson (R-P) isotherm	$q_{e}=\frac{K_{\text{RP}}C_{e}}{1+\alpha_{\text{RP}}{C_{e}}^{\beta_{\text{RP}}}}$	Al-Qodah et al. (2023), Allahkarami et al. (2022), Lee and Ahmad Zaini (2022), Zhao et al. (2023)	4	5
	$\ln\left(\frac{K_{\text{RP}}C_{e}-1}{q_{e}}\right)=\ln\left(\alpha_{\text{RP}}\right)+\beta_{\text{RP}}\;\ln\left(C_{e}\right)$	Jain et al. (2018)	1	
Liu Isotherm	$\ln q_{e}/\left(q_{th}^{e}-q_{e}\right)=n\ln C_{e}-\ln K_{\text{ads}}$ $K_{\text{ads}}={\left(\frac{1}{K_{\text{eq}}}\right)}^{m}$	Thue et al. (2020)	1	1
Sips Isotherm	$q_{e}=\frac{q_{m}K_{s}C_{e}^{1/m}}{1+K_{s}C_{e}^{1/m}}$	Fröhlich et al. (2019), Khalil et al. (2022), Liu et al. (2023)	3	3

As shown in Table 9, the isotherms applied in the literature for the ACMCs include Langmuir (Al-Qodah et al., 2023), Freundlich (Shao et al., 2012), Temkin (Le et al., 2019), Dubinin–Radushkevich (D-R) (Foroutan et al., 2022), the Redlich–Peterson (R-P) (Jain et al., 2018), the Liu (Thue et al., 2020) and the Sips isotherm (Liu et al., 2023). The D-R model is a semi empirical equation in which adsorption follows a pore-filling mechanism. This model assumes a multilayer property involving van der Waals forces and thus is applicable to physical adsorption processes (Foroutan et al., 2022). On the other hand, the R-D isotherm is a compromise between the Freundlich and Langmuir adsorption systems. Finally, the Sip’s isotherm is a combined form of the Langmuir and Freundlich models, developed to estimate the adsorption in heterogeneous systems and it overcomes the high adsorbate concentration limitation associated with the Freundlich equation (Moussa et al., 2021). As shown in Table 7, the most used isotherms for ACMCs are Langmuir and Freundlich isotherms.

### 5.2 Statistical optimization of ACMCs operational parameters

Mathematically, optimization is the search for the best solution under given conditions (constraints) (Singh et al., 2010). Process optimization, in particular, is a fundamental step in designing a cost-effective and efficient process. In many cases, a theoretical model linking some controllable variables or factors to the response is either unavailable or too complex. In this case, information about the relationship between the factors and the response must be obtained experimentally (Box and Wilson, 1951). The traditional approach to optimize process variables requires the determination of a dependent variable in each set of independent variables, then changing only one variable at a time, while keeping all other variables constant. Obviously, this approach requires conducting many experiments, which is time-consuming and expensive approach. Furthermore, it fails to predict the compatibility of operational variables and their effect on the dependent variable. Consequently, various experimental design methodologies have been widely employed to optimize multivariable chemical processes. These methods yield robust statistical models that elucidate the interactive effects among independent process variables and determine their individual influences on the response variable. Furthermore, a significant advantage of this approach is its ability to drastically minimize the number of required experimental runs, thereby reducing both operational time and analytical costs (Singh et al., 2010). Both traditional statistical methodologies and ACMCs machine learning methodologies have been used in the literature for data optimization. The use of statistical factor optimization procedures with multivariate techniques was developed as a fast, economic, efficient, and allow simultaneous optimization of several variables. Among these are the “response surface methodology (RSM)”. The RSM, introduced by Box and Wilson (1951), compromises a set of statistical and mathematical techniques aimed at analyzing problems, using an experimental model, as proposed. The most important benefits of RSM are that the amount of data required for evaluation, analysis, and optimization significantly reduces the total number of tests required, and the response surface is explored with equal rigor and efficiency (Shishir and Chen, 2017). The central component design (CCD) (Box and Wilson, 1951) and the Box-Behnken design (BBD) (Box and Behnken, 1960) are the two most recommended RSM designs for researchers. The first step in RSM is to determine a reasonable estimate of the correct functional relationship between the independent variables and the responses. Typically, a low-order polynomial equation is used for the independent variables. If the response is well explained by a linear function of the independent variable, the chosen function is the first-order model as shown in Equations 1–4 (Sai Datri et al., 2023):

$$y={\;\beta }_{o}+\beta_{1}X_{1}+\beta_{2}X_{2\;}+\beta_{12}X_{1}X_{2}+\varepsilon$$ (1)

If the system has curvature, a higher order polynomial, such as the second-order form, must be followed:

$$y={\;\beta }_{o}+\beta_{1}X_{1}+{\;\beta }_{2}X_{2\;}+{\;\beta }_{1}{X_{1}}^{2}+\beta_{2}{X_{2}}^{2}+\beta_{12}X_{1}X_{2}+\varepsilon$$ (2)

Applying RSM as an optimization tool consists of several steps found in many references (Szpisják-Gulyás et al., 2023).

On the other hand, the “Central Composite Design (CCD)” is widely utilized response surface designs, introduced by Box and Wilson in 1951 (Box and Wilson, 1951). This methodology fits a second-order model to the response variable without requiring a complete three-level factorial experiment. Consequently, a CCD is typically employed when preliminary factorial designs detect curvature in the response data, necessitating an expansion from the initial linear framework to a quadratic response surface design (Singh and Beg, 2013). The total number of experimental runs (N) required to construct a CCD can be determined using the following equation:

$$N=2^{k}+2k+{\;C}_{o}$$ (3)

where *k,* 2^*k*^, 2*k* and *C*_o_ are the number of factors, factorial points, axial/star points and center points. On the other hand, the BBD designs are a class of second-order based on three-level incomplete factorial designs. For three factors, the graph is a cube consisting of a center and edge midpoints, which lie on the surface of a sphere.

The number of experiments (N) required to develop BBD is identified as follows:

$$N=2k\;\left(k-1\right)+{\;C}_{o}$$ (4)

where *k* is a number of factors and *C*_o_ is the number of central points.

In contrast, ACMCs machine learning (ML), a subfield of artificial intelligence (AI), encompasses a suite of computational methods capable of automatically recognizing patterns within datasets to predict future behaviors or guide decision-making under uncertainty (Murphy, 2012). While traditional statistical frameworks necessitate the prior selection of a model that explicitly incorporates theoretical knowledge of the system, ML relies on selecting predictive algorithms based primarily on their empirical performance (Bzdok et al., 2018). Additionally, traditional statistical models offer straightforward interpretability, whereas ML frameworks often operate as ‘black boxes’ that are difficult to interpret, a limitation particularly pronounced in deep neural networks. Furthermore, implementing ML algorithms demands rigorous data preprocessing, intensive training on extensive datasets, and iterative optimization. These techniques are also susceptible to overfitting, a phenomenon where the model fits the training data too closely, thereby compromising its generalizability across independent datasets (Rajula et al., 2020).

Published studies on ACMCs utilize process optimization to refine either the composite’s synthesis parameters or its operational process parameters. As a specific example of synthesis optimization, Jamaludin et al. (2023) employed response surface methodology (RSM) with a central composite design (CCD) across 26 experiments to analyze how the Ni:Fe ratio, the magnetic nanoparticle to granular AC(MNP:GAC) ratio, and the GAC type (self-synthesized vs. commercial) simultaneously impacted the hydrogen production rate (HPR) (Mohd Jamaludin et al., 2023). They determined the optimal conditions to be a Ni:Fe ratio of 0.53 and an MNP:GAC ratio of 0.20 using their self-synthesized GAC, which yielded an HPR of 20.33 ± 0.32 mL H_2_/(L h), a performance 2.78 times higher than the control composite without magnetite binding.

Table 10 lists the studies of ACMCs that have utilized statistical or ML methods to optimize process-operating parameters, which were mainly in adsorptive water treatment applications.

**TABLE 10:** Summary of adsorption operational parameters of some studies using ACMCs.

Composite	Application	Model used	Operating parameters	Predicted responses	Ref.
AC from coconut shell/iron oxide magnetic nanocomposite	To remove Rhodamine B dye	A four-factor (CCD) combined with (RSM) and optimization	T = 50 °C, pH = 2, C_o_ = 220 mg/L, dose = 1 g/L)	A maximum removal (47.44 mg/g) at 1 h equilibrium time of	Singh et al. (2010)
H_2_SO_4_ chemical AC – Fe^0^ – Cu	Removal of CV dye by adsorption	(RSM) and Faced Central Composite Design (FCCD)	C_o_ = 188 ppm, dose = 1.5 g/L, time = 180 min	RE% of 99.501	Abdel-Aziz et al. (2023)
		Artificial neural network (ANN) with Moth search algorithm	C_o_ = 164.4 ppm, dose = 1.03 g/L, time = 170.7 min	RE% of 99.9	
H_3_PO_4_ chemical AC – Fe^0^ – Cu		(RSM) and Faced Central Composite Design (FCCD)	C_o_ = 336 ppm, dose = 1.08 g/L, time = 156 min	RE% of 99.183	
		(ANN) using the algorithm (MSA)	C_o_ = 340.8 ppm, dose = 1.09 g/L, time = 180 min	RE% of 99.9	
Bimetallic AC – Fe^0^ – Cu		(ANN) using the algorithm (MSA)	C_o_ = 148 ppm, dose = 1.3 g/L, time = 180 min	RE% of 99.9	
A magnetic (Fe_3_O_4_) AC from the fruit’s shell, “Brachychiton Populneus”	Removal of MO, Rhodamine B and Phenol	(CCD) coupled with (RSM)	C_o_ at 30 mg/L, dosage at 0.8 g/L, and pH of 4.5	Removal was 92. %, 100. %, and 75% for RhB, MO, and phenol, respectively	Bouider and Rida (2024)

Singh et al. synthesized a coconut shell-derived carbon/iron oxide magnetic nanocomposite and evaluated its effectiveness in removing Rhodamine B (RhB) dye from aqueous solutions (Singh et al., 2010). To maximize removal efficiency, they implemented a four-factor Central Composite Design (CCD) coupled with RSM based on 30 batch adsorption runs. The study optimized four independent operational variables: temperature (10 °C–50 °C), initial pH (2–10), initial dye concentration (140–220 mg/L), and adsorbent dosage (1–5 g/L). For the experimental design, the boundaries were coded into factorial (±1), center (0), and axial (±*α*) points, with the axial distance calculated as *α* = (2^4^)^1/4^. The estimated model coefficients were used to predict response values across various coded variable combinations, with the model’s goodness-of-fit and regression significance evaluated via ANOVA and Student’s t-test using Statistica software. Predictive accuracy was assessed by comparing experimental and predicted responses using *R* ^2^, RMSEP, and RSEP. Under the optimized conditions, pH 2, 50 °C, 220 mg/L dye concentration, and 1 g/L adsorbent dosage, the model’s predicted maximum adsorption capacity of 47.44 mg/g closely aligned with the experimental value of 46.94 mg/g obtained from batch adsorption tests.

Jamaludin et al. (2023) reported a 3D-Surface Model Graph obtained clearly depicted the interaction between two variables (Ni:Fe and MNP:GAC) by keeping the other variable (type of GAC) at the canter point (0) level.

In addition, they reported that each response surface had a clear optimum peak corresponding to the maximum HPR obtained at the center point. Thus, there is a good interaction between the independent variables. The suggested optimum condition of ACMG for bio-hydrogen production were Ni:Fe and MNP:GAC ratios of 0.53 and 0.20, respectively, with GAC-O as the preferable type of GAC. The resulted HPR at these optimal conditions, was 20.33 ± 0.32 mL H2/L. h, 2.78 times higher than the control (GAC without magnetite binding) at only 7.32 ± 0.93 mL H2/L.h. The highest and lowest percentage of hydrogen content in the biogas produced were 64.71%, and 22.25%, respectively.

Abdel-Aziz et al. synthesized fava bean activated carbon-supported bimetallic nano zero-valent iron-copper (AC-Fe^0^-Cu), chemically modified it with either H_2_SO_4_ or H_3_PO_4_, and evaluated its crystal violet (CV) removal efficiency (Abdel-Aziz et al., 2023). To determine optimal conditions, the authors compared two modeling approaches: a 20-run response surface methodology (RSM) quadratic model built in Design-Expert 11, and an artificial neural network (ANN) developed in MATLAB using a sigmoidal-linear transfer function architecture (mapping concentration, dosage, and time to removal efficiency). To maximize performance, the ANN was integrated with the Moth Search Algorithm (MSA) for metaheuristic optimization. Ultimately, this hybrid ANN-MSA approach yielded a higher CV removal efficiency than the standard RSM model. For illustrative purposes, the training, validation, testing, and overall correlation coefficients (R) for the H_2_SO_4_-modified AC(AC–Fe^0^–Cu) system are presented in Figure 9.

**FIGURE 9:**
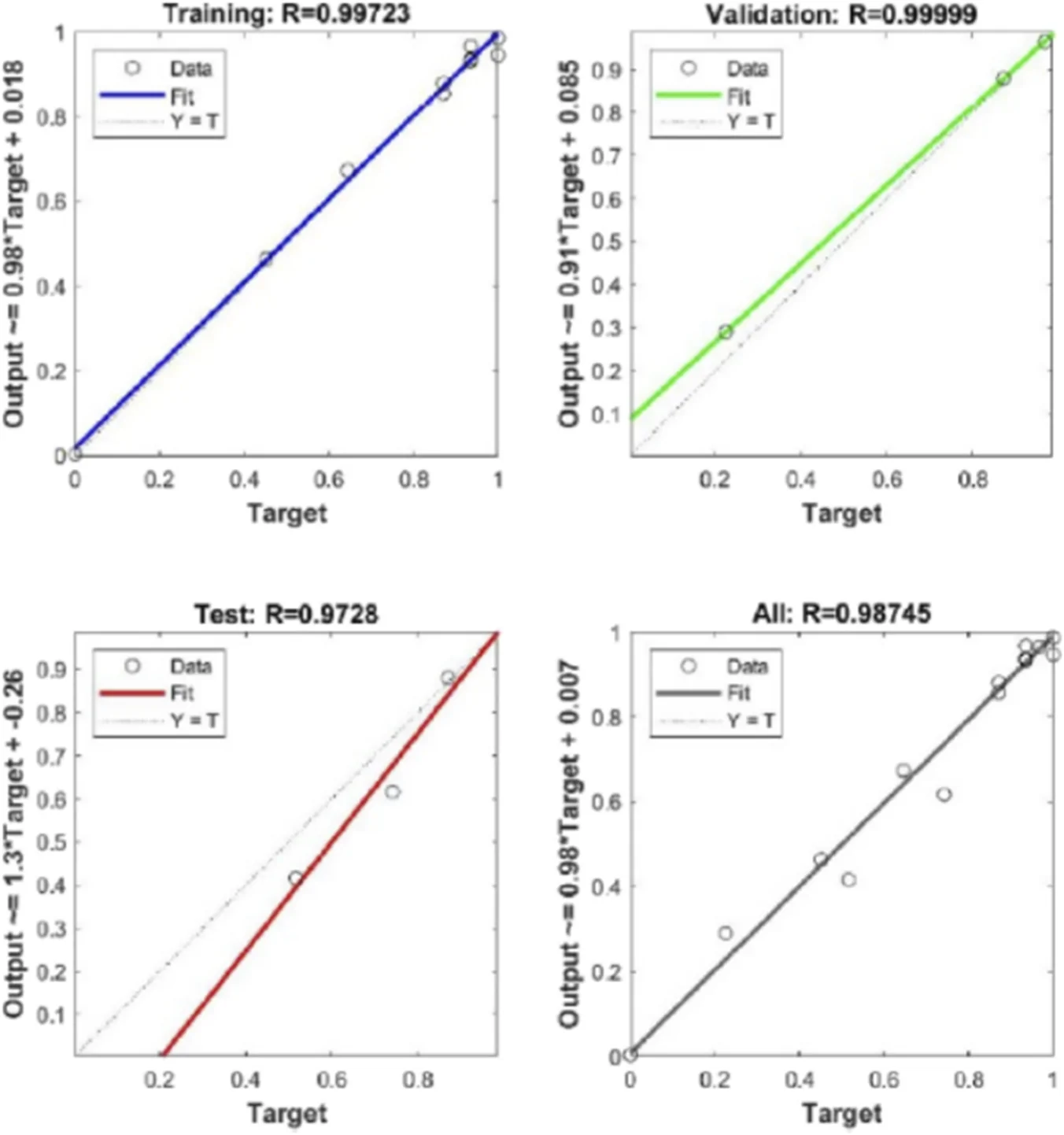
The regression figure for the adsorption of CV by H_2_SO_4_ chemical Fe^0^–Cu. Reprinted with permission from (Abdel-Aziz et al., 2023).

In another study, Boyder et al. fabricated a novel magnetic AC(ACMCs) composite by integrating magnetite (Fe_3_O_4_) with *Brachychiton populneus* fruit shell-derived ACto remove Methyl Orange (MO), Rhodamine B (RhB), and phenol from water (Bouider and Rida, 2024). Using (CCD) with 20 experimental runs, the study optimized three independent operating parameters, initial pollutant concentration (*X*_1_), adsorbent dosage (*X*_2_), and solution pH (*X*_3_), against three corresponding responses: the removal efficiencies of RhB (*Y*_1_), MO (*Y*_2_), and phenol (*Y*_3_). The authors demonstrated that the quadratic response surface model was highly suitable for predicting pollutant removal, supported by strong *R* ^2^ = 0.9128-0.990 for RhB, MO, and phenol removal efficiencies. The identified optimal operating conditions were (*C*_*o*_) of 30 mg/L, dosage of 0.8 g/L, and a pH of 4.5.

To improve prediction accuracy, machine learning methods like Artificial Neural Networks (ANNs) are increasingly used to simulate operational parameters at magnetic composite interfaces. For instance, Deivayanai et al. developed an ANN to model the adsorption of Congo red dye onto a novel magnetic nanoparticle composite synthesized from *Prosopis juliflora* seed waste activated carbon and iron nanoparticles (PJMNPs) (Deivayanai et al., 2024). The network was trained using a backpropagation algorithm with a multilayer perceptron (MLP) architecture, utilizing four input neurons (adsorbent dose, contact time, pH, and temperature) and a single output neuron for dye removal efficiency. The resulting linear fit equation from the validation data can be used to predict output values, as supported by the study’s regression plots and error histograms.

### 5.3 Other models used to describe ACMCs processes

The following paragraphs summarize other models used in the studies of ACMCs that described processes that occur at the surface of ACMCs such as adsorption, electromagnetic waves absorption, surface complexation reactions and desorption or regeneration.

While classical models like the Langmuir and Freundlich isotherms are widely used to describe adsorption on ACMCs, they offer an incomplete understanding due to their simplified assumptions. In contrast, advanced statistical physics models integrate physicochemical parameters that provide detailed, microscopic insights into the mechanism, for example, how much molecules bound per active site, saturation capacity, active site density, and adsorption energy (Akawa et al., 2021; Li et al., 2019; Vaughan and Reed, 2005; Xue et al., 2022). For example, Allahkarami et al. (2023) utilized single and double-layer statistical physical models to study phenol removal using a novel PbFe_2_O_4_ spinel–ACMCs composite compared to pristine activated carbon. Their mechanistic analysis confirmed that the metallic hydroxides within the ACMCs composite significantly enhanced phenol removal by facilitating strong interactions with the pollutant molecules. The authors applied statistical physical modeling to explore the mechanisms responsible for phenol adsorption on MSAC composite and pure activated carbon. The authors demonstrated that these mechanistic studies confirmed the significant role of metallic hydroxides in ACMCs, which is facilitating phenol removal through strong interactions, compared with pristine AC as shown in Figure 10.

**FIGURE 10:**
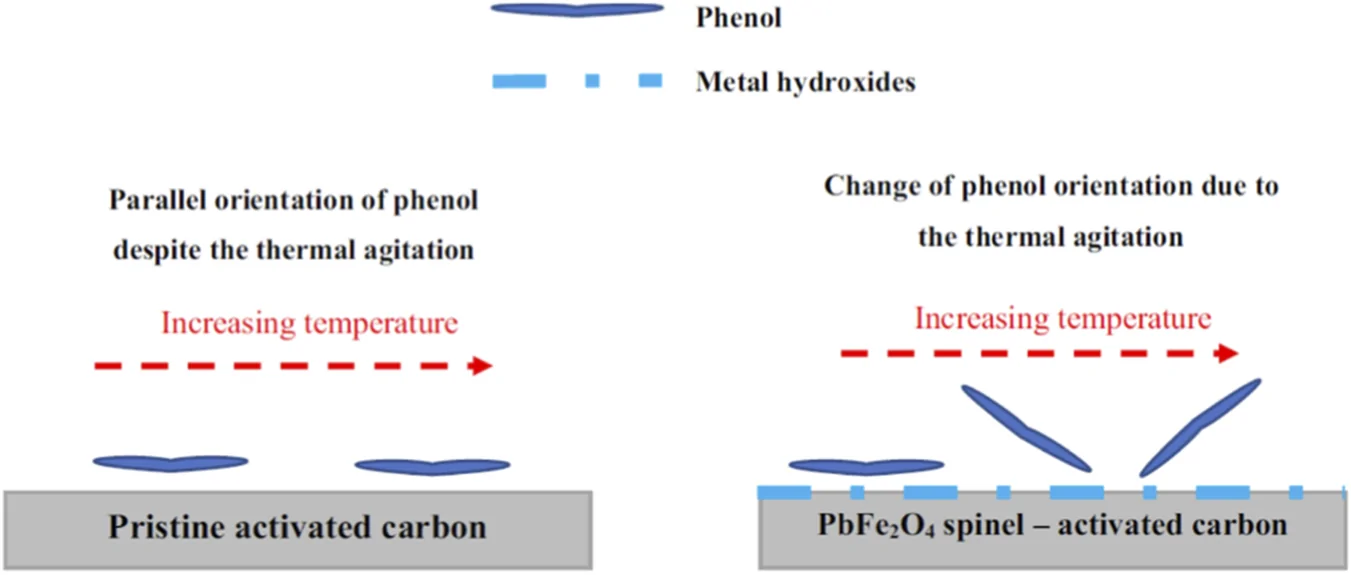
Schematic illustration of the conformational orientation changes of phenol molecules adsorbed onto MSAC and pristine activated carbon induced by thermal agitation. Reprinted with permission from (Allahkarami et al., 2023).

As shown in Figure 10 and based on the monolayer model’s parameters, the geometric orientation of phenol on the magnetic slash-pine activated carbon (MSAC) composite shifted from horizontal (n < 1, where one molecule binds to multiple sites) to inclined (n ≥ 1, where one site anchors multiple molecules) between 298 K and 358 K, driven by thermal agitation and hydrogen bonding. In contrast, phenol on pristine activated carbon (AC) maintained a strictly parallel orientation (n = 0.85–0.99), indicating constrained molecular movement. For both adsorbents, rising temperatures activated new anchoring sites, causing an increase in receptor site density (N_M_). However, this temperature increase also led to a decline in saturation adsorption capacity (Qsat), from 158.94 to 115.23 mg/g for MSAC and from 138.91 to 112.34 mg/g for activated carbon, confirming that the adsorption process for both materials is exothermic.

Zhao et al. synthesized a hierarchical porous magnetic carbon composite (HPMC) by dipping rice in a Ni^2+^ solution and annealing it with solid KOH (Zhao et al., 2019). The researchers optimized the microstructure and composition of the synthesized hierarchical porous magnetic carbon composite (HPMC) to achieve excellent electromagnetic microwave absorbance performance. The authors demonstrated that the microstructure and chemical composition of the HPMC samples could be precisely tailored by simply regulating the mass ratio of KOH/Ni^2+^-treated rice (NTR) powder. Consequently, the synthesized material exhibited excellent electromagnetic microwave absorption performance, achieving a minimum reflection loss (RL_min_) of −52 and a broad effective “absorption bandwidth (EAB)” of 5 GHz at a low filler loading of 15 wt%.

In another study, Vaughan et al. modeled As(V) removal onto iron oxide-impregnated activated carbon (FeAC) utilizing a surface complexation modeling (SCM) approach (Vaughan and Reed, 2005). Mechanistically, simulating inorganic adsorption first requires a mathematical description of the adsorbent surface’s acid-base behavior, operating under the assumption that the specific surface sites responsible for protonation and deprotonation also drive the immobilization of inorganic species. The authors evaluated As(V) adsorption onto FeAC using four distinct surface site–electric double layer (EDL) combinations: (1) diprotic–diffuse layer model (DLM), (2) diprotic–triple layer model (TLM), (3) two-monoprotic–DLM, and (4) two-monoprotic–TLM. Across both EDL frameworks, the two-monoprotic representations yielded superior predictions of the experimental data compared to the diprotic alternatives. Specifically, the two-monoprotic site–TLM accurately described As(V) removal using only two fitting parameters, whereas the diprotic site model required three. They reported the predicted As(V) removal profiles generated by the two-monoprotic– TLM across all FeAC concentrations.

In a separate study, Oh et al. synthesized a magnetic activated carbon composite (CuFe_2_O_4_/AC, ACMCs) that functions as both a dual-functional catalyst for peroxymonosulfate (PMS) activation and a regenerable adsorbent for methylene blue (MB) removal (Oh et al., 2015). To describe the composite’s regeneration rate, the authors developed a kinetic model incorporating simultaneous first-order desorption, second-order adsorption, and pseudo-first-order degradation processes, determining their respective rate constants (*k*_d_, *k*_a_, and *k*_app_) by monitoring MB concentration profiles.

The obtained concentrations profiles showed an initial increase in MB concentration due to desorption, followed by a rapid decline driven by oxidation reactions, proving the material’s efficacy as a regenerable, catalytically active adsorbent. The initial increase in MB concentration is attributed to desorption, which is subsequently followed by rapid oxidation reactions that drive down the MB concentration. Ultimately, their study demonstrated that the ACMCs could effectively serve as a dual-functional catalyst for PMS activation and a regenerable adsorbent.

## 6 Regeneration of ACMCs

Regeneration of adsorbents is a process of cleaning these composites after they have captured the adsorbates or the pollutants. After this regeneration process, the adsorbent restores the adsorption capacity and can be efficiently reused. The application of regeneration procedures for activated carbon magnetic composites (ACMCs) has many key environmental advantages including lowering the cost; preventing secondary waste formation from the expired catalyst; and the sustainable preservation of resources.

Regeneration of ACMCs can be achieved through chemical desorption, thermal treatment, or advanced oxidation processes (AOPs), taking advantage of the magnetic core for rapid solid-liquid separation.

Regeneration of ACMCs can be achieved through by various methods, taking advantage of their magnetic properties to facilitate separation from the treated solution. The chemical regeneration method is the most common technique, utilizing acidic or alkaline regenerants. Among acidic reagents used to regenerate ACMCs, 0.1 M HCl was the most frequently used to serve that purpose (Jain et al., 2018; Kakavandi et al., 2015; Moussa et al., 2021; Reza et al., 2015), meanwhile, NaOH alkaline regenerant was mainly employed with concentrations ranging from 0.05 M to 2 M (El Meziani et al., 2025; Gheitasi et al., 2022; Ngan et al., 2024; Rafli et al., 2021; Rinovian et al., 2026; Wen et al., 2022; Zarei and Sinapour, 2015). A few studies combined alkaline desorption followed by an acid treatment step to enhance the efficiency of the regeneration process (Mirzaee, 2022).

A thermal regeneration approach was used to regenerate ACMCs via heating the spent adsorbent. This approach was found to restore the capacity of ACMCs, however, slightly mass loss was observed after repeated cycles (Lee and Ahmad Zaini, 2022; Luu et al., 2022). Oxidative regeneration represents an alternative technique for MAC, where a peroxymonosulfate reagent was used to restore adsorption activity through oxidation of adsorbed pollutents (Oh et al., 2015). In addition to previous techniques, photo-Fenton method was also reported as another option for ACMCs regeneration. In this method, hydrogen peroxide in conjunction with UV irradiation was applied to simultaneously degrade the adsorbed pollutants and regenerate the adsorbent surface (Molina-Jordá, 2022). Burning of spent adsorbent (Mirzaee and Sartaj, 2022) and solvent extraction (Saning et al., 2024) techniques were also utilized to regenerate ACMCs, however, less frequently.

The regenerated ACMCs exhibited good regeneration stability, where stable adsorption performance was reported over four to seven regeneration cycles (El Meziani et al., 2025; Gheitasi et al., 2022; Jain et al., 2018; Kakavandi et al., 2015; Luu et al., 2022; Mirzaee, 2022; Molina-Jordá, 2022; Rafli et al., 2021; Rinovian et al., 2026; Saning et al., 2024; Wen et al., 2022). The majority of these attempts retain approximately 80% of the original adsorption capacity values after repeated regeneration cycles (Gheitasi et al., 2022; Rinovian et al., 2026; Wen et al., 2022).

Most studies have characterized the freshly synthesized ACMCs using SEM, FTIR, XRD, BET, TEM, VSM techniques, however, a few studies repeated these characterizations after several regeneration cycles in order to evaluate deterioration or alternation in their magnetic properties (El Meziani et al., 2025; Gheitasi et al., 2022; Jain et al., 2018; Rafli et al., 2021). Therefore, long-term stability and degradation mechanisms of MAC are not fully understood.

Overall, few researches have considered regeneration of adsorbents including activated carbon magnetic composites (Barzegarzadeh and Amini-Fazl, 2025a; Iles et al., 2025; Mirzaee and Sartaj, 2022; Oh et al., 2015; Song et al., 2023; Spessato et al., 2020). For this reason, it is recommended in intensify such researches to increase the number of adsorption cycles and reduce the cost of such adsorbents.

## 7 Conclusion

It is evident from the literature survey, which shows an increasing number of publications on ACMCs that these materials are emerging as promising candidates for many applications including mainly as adsorbents for pollutants and environmental protection. At the same time, these magnetic composite materials are easy to prepare and separate, which has a good effect in practical applications. In addition, ACMCs materials have proven to be effective adsorbents for both organic and inorganic common pollutants found in industrial wastewater and in polluted air.

ACMCs have proven to be materials of great interest in many applications. Researches published since the year 2000 have shown that the adsorption capacity of these composites significantly increased compared to standalone activated carbon. In addition, the presence of magnetic materials in the activated carbon particles enhanced their selectivity and consequently the removal efficiencies. Most adsorption applications were to remove organic pollutants mainly dyes industrial wastewater. In addition, ACMCs have been used as catalysts and biocatalyst support. Enzymes and whole cells were successfully immobilized in on the surface of these composites, which then used as efficient biocatalysts in bioreactors.

In addition, it seems that there is no complete common system for the synthesis technology of such composite materials. For this reason, the quality of the products are very different. Accordingly, in the future of the practical preparation, various factors affecting the preparation of ACMCS should be considered, and more accurate synthesis processes should be explored.

## 8 Challenges, perspectives and future recommendations

Although ACMCs has made significant progress mainly in pollutant treatment. However, there are still many challenges concerning the use of ACMCs composites as efficient adsorbents. Accordingly, future researches should consider the following recommendations:

- The stability, long-term use and the possibility of regeneration of these ACMCs should be more tested to standardize the most efficient preparation procedures (Shan et al., 2026; Sun et al., 2025).
- More researches are needed to treat real pollutants rather than synthetic or simulated wastewater especially those containing organics-water emulations and industrial mixtures of heavy metal ions wastewater.
- Since most of the researches are still applying, lab-scale batch processes that test limited number of parameters affecting the process. Continuous processes in both lab and pilot scale are needed to obtain more suitable results for scale-up into industrial scale applications.
- Moreover, it seems that there is no complete common system for the synthesis technology of such composite materials and the quality of the products are very different. For this reason, the quality of the products are very different.
- The regeneration of ACMCs is a very necessary cost-effective step after it becomes saturated with the adsorbate. However, very limited number of researches apply regeneration procedure to test the ACMCs activity after several adsorption desorption cycles. For this reason, more regeneration researches are needed.
- Since the adsorption processes produces solid wastes that include the expired adsorbent, the “zero waste” approach should be advanced to solid waste management (Li et al., 2026).

Finally, we hope that this review can provide some ideas for the development and application of ACMCs magnetic composites. It is believed that these composites will become efficient and cost effective environmental protection materials and catalysts in the near future.

## Glossary

***q***_***t***_: The adsorption capacity of the adsorbate at time *t* (mg/g)
***q***_***e***_: The adsorption capacity of the adsorbate at equilibrium (mg/g)
***k***_***1***_: The pseudo-first-order rate constant of adsorption (min^−1^)
***k***_***2***_: The pseudo-second-order rate constant of adsorption (g mg^−1^ min^−1^)
**V**_**o**_: Initial velocity (moles/time)
**V**_**max**_: Maximum velocity
**S**: Substrate concentration (molar)
**K**_**m**_: Substrate concentration at half V_max_
***V***: The volume of the crystals
***V***_**0**_: The initial crystal volume (usually zero)
***V***_**m**_: The maximal crystal volume attained after crystallization is complete
***K***: A temperature-dependent constant (similar to a rate constant)
***n***_**AV**_: The Avrami exponent (which reflects the dimensionality of “crystal” growth)
***Α***: The chemisorption rate at zero coverage (mg g^−1^ min^−1^)
***Β***: The desorption rate constant (mg g^−1^ min^−1^)
***k***_***i***_: The intraparticle diffusion rate constant (mg g^−1^ min^−1/2^)
***C***_***i***_: A constant related to the thickness of the boundary layer; the larger the value of *C*_*i*_, the greater is the effect of the boundary layer
***B***_**t**_: A mathematical function of F
***F***: The ratio of amount of the adsorbed solute (mg/g) at any time *t* and at equilibrium
$\bar{q}$: The average adsorbate adsorption capacity
***q*** ^*******^: The adsorbate adsorption capacity related to the concentration in the liquid phase at equilibrium (mg/g^−1^)
***k***_**LDF**_: The LDF mass transfer coefficient (1/min)
**K**: A constant (mg/g)
**V**: A positive constant (<1) (min^−1^)
***C***_***e***_: The adsorbate concentration at equilibrium (mg/L)
***K***_**L**_: Langmuir isotherm constant related to the affinity of the adsorbate with the adsorbent (L/mg)
***q***_***m***_: A constant stands for the maximum adsorption capacity (mg/g)
***a***_**L**_: A Langmuir constants related to the adsorption energy
***K***_**F**_: The Freundlich isotherm constant (mg(^1−1/*n*^) L^(1/*n*)^ g^−1^)
***N***: A constant related to the adsorption intensity and the degree of heterogeneity of the adsorbent surface
***β***_**t**_ **, *K***_**T**_ **, *b***_**T**_: Constants of Temkin isotherm model
***R***: The gas constant
***T***: The absolute temperature
***β***_**DR**_: A constant related to the adsorption energy(mol^2^/J^2^)
ε: A constant calculated from the $\left(\varepsilon =RT\;\ln\left(1+\frac{1}{C_{e}}\right)\right)$ equation
***E***: The mean free energy of adsorption (kJ/mol)
***K***_**RP**_ ***, α***_**RP**_ ***, β***_***RP***_: The Redlich–Peterson constants
***K***_**ads**_: A constant related to the magnitude of the thermodynamic equilibrium constant, *K*_eq_
$q_{th}^{e}$: The theoretical amount that could be adsorbed by unit adsorbent at equilibrium
***M***: A constant in Liu isotherm
$K_{s}$: The adsorption equilibrium constant of the Sips equation
***1/m***: A constant used to measure the heterogeneity of the adsorption sites on the surface of the adsorbent. When the *m* value is uniform, the Sips isotherm can be regarded as a traditional Langmuir isotherm
